# Effects of breakfast macronutrient composition on postprandial glycemic control in patients with diabetes mellitus: a systematic review and meta-analysis of randomized controlled trials

**DOI:** 10.3389/fendo.2026.1859725

**Published:** 2026-08-17

**Authors:** Jing-Xian Fang, Fang Huang, Qing Gu, Jian Meng, Hui-Ming Zou, Yu Han, Yue-Xia Han, Hui-Zhen Liu, Qian-Wen Ma, Xi-Shuang Chen, Sui-Jun Wang

**Affiliations:** Yangpu District Shidong Hospital of Shanghai, Endocrinology and Metabolism, Shanghai, China

**Keywords:** breakfast, diabetes mellitus, macronutrient and dietary fiber composition, network meta-analysis, postprandial glycemic control, randomized controlled trials

## Abstract

**Background:**

As the first meal of the day, breakfast significantly impacts the daily blood glucose profile of people with diabetes. However, there is currently no consensus on the optimal macronutrient composition of breakfast (i.e., the appropriate ratio of carbohydrates, protein, fat, and dietary fiber). This systematic review and network meta-analysis aims to evaluate the effects of different macronutrient compositions in breakfast on postprandial blood glucose control in people with diabetes.

**Methods:**

We searched the PubMed and Web of Science databases for studies published from the inception of each database through March 23, 2026. We included randomized controlled trials comparing the effects of different breakfast macronutrient compositions on glucose-related outcomes in patients with diabetes. Two researchers independently conducted literature screening, data extraction, and risk-of-bias assessments (using the RoB 2.0 tool). We conducted pairwise and network meta-analyses using a random-effects model. Effect sizes were reported as standardized mean differences or mean differences with 95% confidence intervals. Interventions were ranked using the SUCRA score.

**Results:**

A total of 25 randomized controlled trials were included. Compared with a standard breakfast, adjusting the macronutrient composition of breakfast significantly reduced 2-hour postprandial blood glucose (mean difference = −1.90 mmol/L; 95% confidence interval: −2.46 to −1.34; P< 0.001), glycated hemoglobin (mean difference = −0.52%; 95% confidence interval: −0.59% to −0.45%; P< 0.001), and the area under the postprandial glucose curve (standardized mean difference = −1.24; 95% confidence interval: −2.45 to −0.03; P = 0.045). Subgroup analyses showed that legume-based breakfasts and low-glycemic-index breakfasts were most effective in reducing 2-hour postprandial blood glucose, while breakfasts supplemented with whey protein were most effective in improving HbA1c. Network meta-analysis and SUCRA ranking indicated that legume-based breakfasts (85.7%) and low-glycemic-index breakfasts (71.4%) were the preferred breakfast choices. No significant publication bias was detected, and the results of sensitivity analyses were robust.

**Conclusion:**

Adjusting the macronutrient and dietary fiber composition of breakfast can significantly improve postprandial blood glucose control in people with diabetes. Legume-based breakfasts and low-glycemic-index breakfasts were most effective at lowering 2-hour postprandial blood glucose levels, while breakfasts supplemented with whey protein were most effective at reducing HbA1c.

**Systematic Review Registration:**

https://www.crd.york.ac.uk/PROSPERO/, identifier CRD420261367773.

## Introduction

Diabetes is one of the most common chronic metabolic diseases worldwide and has become a serious public health issue (1). Studies indicate that hyperglycemia is an independent risk factor for cardiovascular complications and all-cause mortality in diabetes (2).

The DECODE study demonstrated that 2-hour postprandial glucose levels are more strongly associated with cardiovascular mortality than fasting glucose levels (3). Therefore, in diabetes management, attention should be focused not only on long-term glycemic indicators such as fasting glucose and glycated hemoglobin (HbA1c) but also on postprandial glucose control.

As the first meal of the day, breakfast significantly impacts the daily blood glucose profile (4). Epidemiological evidence suggests that regular breakfast consumption improves glycemic control (5). A cross-sectional study of a Spanish population (n=514) using continuous glucose monitoring found that the glycemic load of breakfast was directly associated with postprandial glucose levels (6). In contrast, breakfasts consisting primarily of traditional foods such as whole grains were associated with a lower risk of hyperglycemia. A cohort study of Chinese adults found that traditional wheat-based breakfasts were negatively associated with the risk of new-onset hyperglycemia (OR = 0.67) (7).

Breakfast is also a key signal for resetting the body’s internal “clock” and preparing for the “dawn phenomenon.” Its physiological significance is reflected in a finely tuned regulatory network involving the brain, hormones, and genes (8). A multinational project called the “International Breakfast Research Initiative” analyzed data from national nutrition surveys in the United States, Canada, and several European countries. The results showed that in these countries, the energy people actually consume at breakfast accounts for 16% to 21% of their total daily energy intake (9).

All RCTs included in this study used a “standard breakfast” or an “isocaloric control breakfast” as the control group, rather than “skipping breakfast”; therefore, the conclusions of this study apply to macronutrient optimization under the premise of “eating breakfast” and do not involve a comparison of whether or not breakfast is consumed.

However, there is currently no consensus on the optimal macronutrient composition of breakfast—that is, the appropriate balance of carbohydrates, protein, fat, and dietary fiber. Nevertheless, study results remain inconsistent due to significant variations in intervention types, outcome measures (2-hour blood glucose, AUC, iAUC, HbA1c, etc.), sample sizes, and intervention durations across studies. More importantly, there are currently no systematic reviews or meta-analyses that comprehensively compare and rank different breakfast macronutrient compositions.

Traditional pairwise meta-analyses can only compare two interventions and cannot simultaneously compare and rank multiple interventions. Network meta-analysis (NMA) integrates evidence from both direct and indirect comparisons to quantitatively compare and rank the effects of multiple interventions, thereby providing a more comprehensive evidence-based foundation for clinical decision-making (10). In recent years, the SUCRA (Surface Under the Cumulative Ranking) score has been widely used to rank interventions, with higher values indicating better efficacy.

This systematic review and meta-analysis aims to evaluate the effects of different macronutrient compositions of breakfast on postprandial blood glucose control in patients with diabetes.

## Methods

### PICO

Population (P): Adults (≥18 years) diagnosed with Type 1, Type 2, or Gestational Diabetes Mellitus.

Intervention (I): Breakfasts with adjusted macronutrient composition (e.g., low-glycemic-index, high-fiber, low-carbohydrate, whey protein-supplemented, legume-based).

Comparison (C): Standard breakfast, isoenergetic control breakfast, or regular breakfast.

Outcomes (O): 2-hour postprandial glucose (2h-PPG), glycated hemoglobin (HbA1c), area under the curve (AUC), incremental AUC (iAUC), etc.

### Protocol and guidance

This systematic review and meta-analysis were conducted and reported in accordance with the Preferred Reporting Items for Systematic Reviews and Meta-Analyses (PRISMA) guideline. The study protocol has been registered with PROSPERO (registration number: CRD420261367773).

### Data sources, search strategy, and definitions

We conducted a literature search in the PubMed, Cochrane CENTRAL, ScienceDirect, Embase, and Web of Science (WOS) databases, covering the period from the inception of the databases through March 23, 2026. Search terms included “diabetes,” “Breakfast Macronutrient Composition,” and “Postprandial Glycemic Control.” We expanded the search scope using relevant synonyms, covering title, abstract, and keyword searches across all databases. After the search was completed, all literature records were imported into EndNote reference management software, and duplicates were removed using automated tools. Subsequently, two researchers independently conducted an initial screening of titles and abstracts and a secondary screening of full texts based on predefined inclusion and exclusion criteria (see the “Literature Screening Criteria” subsection below for details). Any disagreements were resolved through discussion or consultation with a third researcher. The complete search strategy is detailed in **Supplementary Table 1** of the **Supplementary Material**.

### Study selection: inclusion and exclusion criteria

The study population included patients diagnosed with diabetes mellitus, including type 2 diabetes mellitus (T2DM), type 1 diabetes mellitus (T1DM), and gestational diabetes mellitus (GDM). Eligible participants were adults aged 18 years or older, regardless of gender, race, disease duration, or treatment regimen. Diagnosis of diabetes was based on the criteria established by the American Diabetes Association (ADA), the World Health Organization (WHO), or national diabetes management guidelines. While the original studies did not all require participants to have a regular breakfast routine, all of them required participants to eat breakfast on the day of the trial. We explicitly stated that we included studies regardless of intervention duration (acute single-meal studies to long-term trials).

In terms of interventions, adjustments to the macronutrient composition of breakfast include, but are not limited to: low glycemic index (Low GI) breakfasts, high dietary fiber breakfasts, low-carbohydrate breakfasts, whey protein supplement breakfasts, bean-based breakfasts, cranberry supplements, cocoa supplements, and breakfast alternatives (e.g., specialized nutritional formulas for diabetes). Control measures include: standard breakfast, isoenergetic control breakfast, placebo control, and regular breakfast. Report at least one of the following glucose-related outcome measures: 2-hour postprandial glucose (2h-PPG), area under the curve (AUC), incremental area under the curve (iAUC), partial area under the curve (Partial AUC, pAUC), glycated hemoglobin (HbA1c), fasting plasma glucose (FPG), blood glucose peak, time in range (TIR), and mean blood glucose (MBG). All included studies were randomized controlled trials (RCTs).

Exclusion criteria include: non-English studies; observational studies; studies with fewer than 5 participants per group; non-randomized controlled trials (such as observational studies, case reports, reviews, conference abstracts, commentaries, and letters); studies involving non-diabetic participants (such as healthy individuals or those with prediabetes); studies that do not report relevant glycemic outcomes; and studies from which effect sizes (mean, standard deviation, sample size) cannot be extracted or calculated.

**Figure 1:**
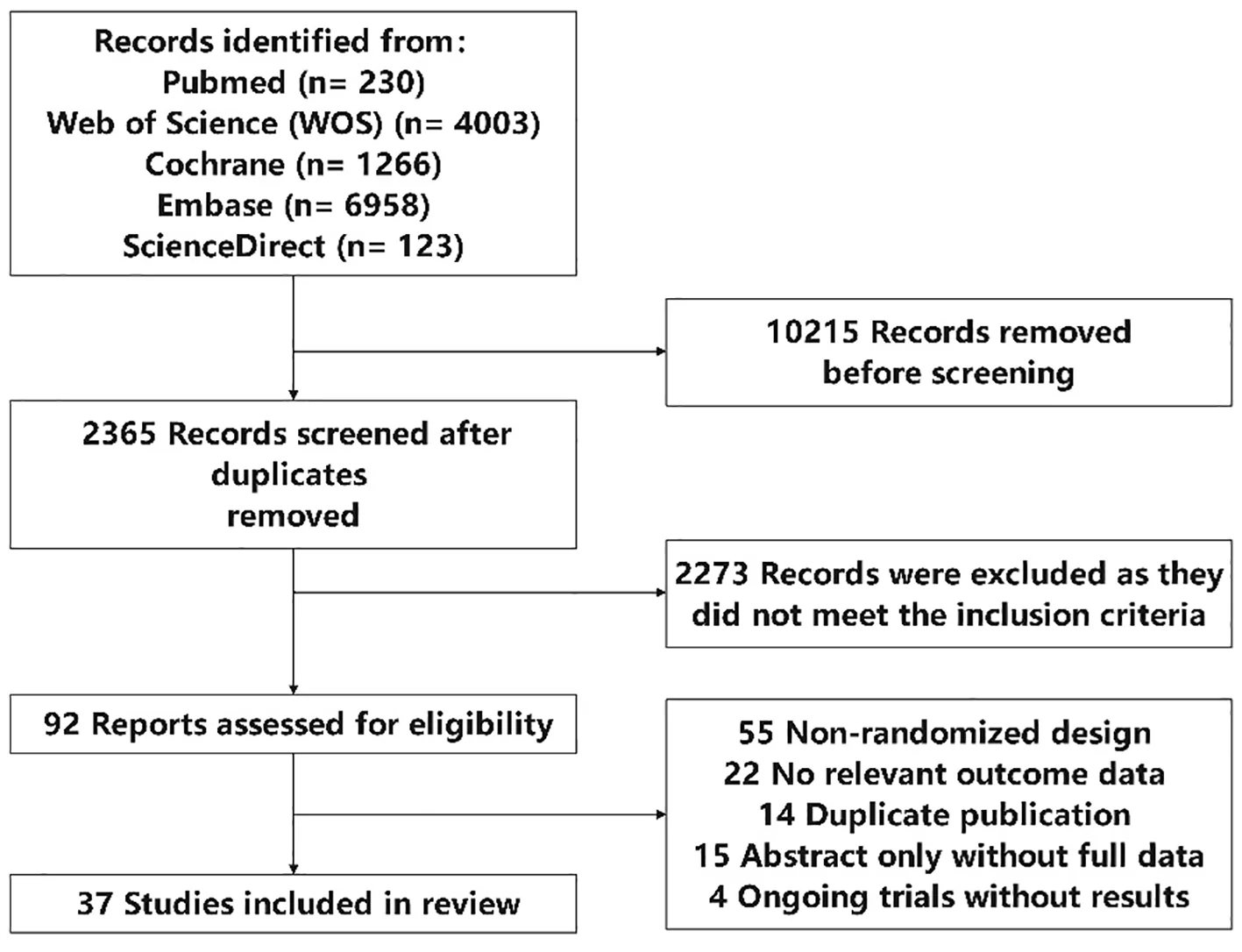
Flowchart of studies.

Two researchers independently conducted the literature screening. First, duplicate references were removed using EndNote software; then, a preliminary screening was performed by reviewing titles and abstracts to exclude studies that clearly did not meet the inclusion criteria; finally, a secondary screening was conducted by reviewing the full texts to determine which studies were ultimately included. Any disagreements during the screening process were resolved through discussion and consensus, with a third researcher making the final decision when necessary. The literature screening process and reasons for exclusion are detailed in the PRISMA flowchart (**Figure 1**).

### Risk of bias assessment

The Cochrane RoB 2.0 tool was used to assess the risk of bias in the included studies. The domains assessed included: the randomization process, deviation from the intended intervention, missing outcome data, outcome measurement, and selective reporting. The risk of bias for each domain and overall was categorized into three levels: “low risk of bias,” “moderate risk of bias,” and “high risk of bias.” Two researchers conducted independent assessments, and any disagreements were resolved through discussion or third-party arbitration. The results were visualized using a summary risk of bias plot, and a sensitivity analysis was performed based on the assessment results (excluding studies with a high risk of bias).

### Statistical analysis

All statistical analyses were performed using R version 4.6.0 with the meta and metafor packages.

This study employed network meta-analysis (NMA) within a frequency-based framework to conduct indirect and mixed comparisons of various interventions.

To rank the effectiveness of interventions, this study will calculate the Surface Under the Cumulative Ranking Curve (SUCRA). SUCRA is a quantitative metric that provides a comprehensive ranking of each intervention, with values ranging from 0 to 1 (or converted to percentages from 0% to 100%). Its clinical significance lies in the fact that a higher SUCRA value indicates greater certainty that the intervention ranks higher in the hierarchy and a higher posterior probability that it is the optimal choice.

For continuous outcomes, the standardized mean difference (SMD) with 95% confidence intervals (CIs) was calculated. SMD values of 0.2, 0.5, and 0.8 were considered small, moderate, and large effects, respectively. For binary outcomes, the relative risk (RR) with 95% CIs was estimated.

Intention-to-treat (ITT) data were preferentially extracted when available. When ITT data were not reported, per-protocol or completer analysis data were used. For binary outcomes, the total number of randomized participants was used as the denominator, strictly adhering to the ITT principle.

Heterogeneity was assessed using the Cochran’s Q test and the I^2^ statistic. And I^2^ value< 50% with a Q test P > 0.10 indicated acceptable heterogeneity, and a fixed-effects model was applied. Conversely, I^2^ ≥ 50% or Q test P ≤ 0.10 indicated substantial heterogeneity, warranting the use of a random-effects model (Der Simonian-Laird method). All statistical tests were two-tailed, with P< 0.05 considered statistically significant.

Publication bias was evaluated using a combined approach: (1) visual inspection of funnel plot symmetry; (2) Egger’s regression test for continuous outcomes and Harbord’s test for binary outcomes; and (3) trim-and-fill analysis to adjust for potential asymmetry.

To assess the robustness of the findings, the following sensitivity analyses were conducted: (1) leave-one-out analysis (iteratively removing each study and recalculating the pooled effect); (2) quality-based sensitivity analysis (excluding studies with high risk of bias); and (3) small-study sensitivity analysis (excluding studies with small sample sizes, e.g., total n< 20).

### Definitions

Standard breakfast: A control meal representing typical macronutrient distribution (e.g., 50-60% carbohydrates, 15-20% protein,<30% fat) based on local dietary guidelines or the original study’s definition.

Low-glycemic-index (GI) breakfast: Breakfast with GI value ≤ 55.

High-fiber breakfast: Breakfast containing ≥ 5g of dietary fiber per serving.

iAUC: Incremental area under the curve, calculated using the trapezoidal method, excluding baseline glucose levels.

## Results

### Study characteristics

A total of 37 studies were included in this systematic review, comprising 1,030 participants with type 2 diabetes mellitus (T2DM) (11–42), gestational diabetes mellitus (GDM) (43–45), or type 1 diabetes mellitus (T1DM) (46, 47). The studies were published between 1984 and 2025, with the majority conducted in the last two decades (2000–2025: n = 27, 73.0%).

### Intervention characteristics

Interventions were categorized into five main types based on their primary mechanistic approach: (1) high-protein breakfasts (eggs, whey protein, lupin-enriched foods); (2) high-fiber breakfasts (whole grains, legumes, viscous fiber, bran); (3) low-glycemic index (low-GI) breakfasts; (4) low-carbohydrate/high-fat breakfasts; and (5) supplement-based interventions (flavonoids, functional ingredients). The basic characteristics of each study are shown in **Tables 1**, **2**.

**Table 1:** Characteristics of Included Studies.

Author (country, year of publication)	Population	Sample size	Age (years; mean (SD)*)	Intervention group (breakfast description)	Intervention breakfast macronutrients (g, % kcal)	Control group (breakfast description)	Control breakfast macronutrients (g, % kcal)	Isocaloric design	Key intervention dose (g)	Assessment of exposure	Outcome measures	Duration of study
Li Wenhu (China, 2008)	Male Han Chinese with T2DM	9	NR	High-Fat Diet (HFD) (3-day isocaloric standard diet)	Fat: 50% of total kcal Carb: 35% of total kcal Protein: 15% of total kcal (Total energy: 8786.4 kJ/d/2100 kcal)	High-Carbohydrate Diet (HCD) (3-day isocaloric standard diet)	Fat: 25% of total kcal Carbohydrate: 60% of total kcal Protein: 15% of total kcal (Total energy: 8786.4 kJ/d/2100 kcal)	Yes	NR	Eat under supervision	Fasting Plasma Glucose (FPG)	3d
Ballesteros (Mexico, 2015)	T2DM	29	53.5 ± 8.3	One Egg Breakfast (1 large egg, usually scrambled, with vegetables + 2 slices bread or 2 tortillas)	Provided by egg: Protein: 8g Fat: 6.8 Carb: 0.3g Chol: 250mg (Egg weight ~65g) Total breakfast (self-reported): ~313 kcal/day	Oatmeal Breakfast (40g Quaker oatmeal + 472 mL lactose-free milk)	Provided by oatmeal + milk: Protein: 5.5g Fat: 3.6g Carbs: 23.6g Fiber: 3.2g (0.85g soluble) Total breakfast (self-reported): ~335 kcal/day	Yes	1egg/day (approx. 65g, providing 250mg cholesterol)	Provide physical items + verification of return	Fasting Plasma Glucose (FPG)	35d
Golay (Switzerland, 1992)	T2DM	14	67 ± 11	Muesli Breakfast (SRS - slow release starch) (65g muesli + 120mL whole milk)	Total breakfast (per serving): Carb: 46g - Starch: 26g - Sugars: 20g Protein: 11g Lipid: 6.2g Dietary fibre: 6.7g Energy: ~1200 kJ (~287 kcal)	Cornflakes Breakfast (FRS - fast release starch) (35g cornflakes + 12g sugar + 120mL whole milk)	Total breakfast (per serving): Carbohydrate: 46g - Starch: 26g - Sugars: 20g Protein: 7g Lipid: 4.4g Dietary fibre: 1.2g Energy: ~1080 kJ (~258 kcal)	Yes	65g muesli (providing slow-release starch from raw rolled wheat and white bean flakes)	Food Diary	Mean daytime blood glucose	14d
Narayana (India, 2016)	T2DM	105	49.3 ± 9.9	Foxtail Millet Dosa (unpolished foxtail millet + black gram, fermented batter)	Per 50g available Carb serving: Protein: 15g Fat: 4.0g Minerals: 3.5g Total Carbohydrate: ~72g * Total Dietary Fibre: 20.7g Energy: 354 kcal	Rice Dosa (polished rice + black gram, fermented batter)	Per 50g available CHO serving: Protein: 7.3g Fat: 0.4g Minerals: 0.9g Total Carbohydrate: ~52.9g * Total Dietary Fibre: 4.2g Energy: 267 kcal	Yes	290g foxtail millet batter (providing 50g available carbohydrate)	Eat under supervision	2-hour postprandial glucose (2h-PPG)	1d
Lopez-Romero (Mexico, 2014)	T2DM	14	48 ± 2.1	HSPB + Nopal (High-Soy-Protein Breakfast + 300g steamed nopal)	HSPB (base): 344 kcal, 42.4% Carb, 40.7% protein, 16.9% fat (soy hamburger 61.5g + soy milk beverage 230mL) + Nopal: 300g raw (~250g cooked)	HSPB alone	HSPB: 344 kcal, 42.4% CHO, 40.7% protein, 16.9% fat	Yes	300g raw nopal (steamed)	Eat under supervision	iAUC	1d
Clark (USA, 2006)	T2DM	45	Males: 64 ± 2; Females: 59 ± 3	Breakfast B (Low-Glycemic Load + Psyllium fiber) Psyllium RTE cereal (2 servings) + skim milk + wheat toast + margarine + coffee/tea	Per breakfast: Energy: ~1515 kJ (~362 kcal) Carb: 62g Protein: 19g Fat: 6.0g Total dietary fiber: 12.4g Soluble fiber: 6.6g (GI of cereal: 56, white bread reference)	Breakfast A (High-Glycemic Load, no psyllium) Farina (2 servings) + skim milk + wheat toast + margarine + coffee/tea	Per breakfast: Energy: ~1833 kJ (~438 kcal) Carbohydrate: 78g Protein: 19g Fat: 5.5g Total dietary fiber: 3.4g Soluble fiber: 1.0g	No	6.6g soluble fiber (from psyllium RTE cereal) + lower glycemic load (38% lower than Breakfast A)	Standardized Meals + Frequent Blood and Biochemical Monitoring	Log AUC Glucose (AM)	1d
Kabir (France, 2002)	T2DM	13	59 ± 2	Low-GI Breakfast (Low-GIB) Muesli (oat bran concentrate, apple, fructose) + pumpernickel bread + milk + butter	Per breakfast (~20% daily energy): Energy: ~263 kcal Availabl Carb: 37.4g Protein: 9.5g Fat: 8.3g Total fiber: 10.2g β-glucan: 3.0g (from oat bran) Estimated GI: 40%	High-GI Breakfast (High-GIB) Whole wheat cereal (Weetabix) + wholemeal wheat bread + milk + butter	Per breakfast (~20% daily energy): Energy: ~249 kcal Available CHO: 37.0g Protein: 9.0g Fat: 6.9g Total fiber: 8.7g β-glucan: 0g Estimated GI: 64%	Approximately isocaloric	3g β-glucan (from oat bran in muesli) + low-GI carbohydrate sources (pumpernickel bread GI 41%, muesli GI 41%)	Food Diary + Follow-up with a Nutritionist + Standardized Meals	HbA1c	28d
Pearce (Australia, 2008)	T2DM	23	61.0 ± 10.0	CARB-B (Breakfast-loaded) Carbohydrate concentrated at breakfast (~125g), identical foods as other treatments	Per day (9 MJ, ~2150 kcal) Carb: 40% (~215g) Protein: 34% (~180g) Fat: 26% (~62g) Breakfast: ~128g Carb (of daily total)	CARB-E (Even distribution) Carb evenly distributed across 3 meals (~70g each)	Per day (9 MJ, ~2150 kcal): Carb: 40% (~215g) Protein: 34% (~180g) Fat: 26% (~62g) Each meal: ~70g Carb	Yes	~125g carb in the “loaded” meal (breakfast, lunch, or dinner) vs ~70g in evenly distributed treatment	Complete food records + weighed food diary + continuous glucose monitoring (CGMS)	Daily Gmax (mmol/L)	3d
Ward (Australia, 2020)	T2DM	22	58.0 ± 6.6	Lupin-Enriched Foods (daily consumption of lupin-containing bread, pasta, Weetbix™ cereal, breadcrumbs at breakfast+lunch daily, dinner ≥3d/wk)	Estimated total daily diet: Energy: 4164 kJ/d Protein: 71g/d Carb: 96g/d Fat: 29g/d Fibre: 29g/d (Lupin provided ~20% daily energy)	Energy-Matched Control Foods (wheat-based: bread, pasta, Weetbix™ cereal, breadcrumbs at same meal frequency)	Estimated total daily diet: Energy: 4674 kJ/d Protein: 62g/d Carb: 140g/d Fat: 27g/d Fibre: 22g/d	Yes	~45g lupin kernel flour/day (providing ~12g protein + ~10g fibre/day)	Food Diary + Food Frequency Questionnaire	Mean home blood glucose (mmol/L)	56d
Jenkins (Canada, 2002)	T2DM	23	63 ± 1	High-Fiber Test Phase High-wheat bran bread and breakfast cereal provided daily (~24% daily energy)	From supplements (per day): Energy: ~24% of total daily intake Fat: 5.9% energy Protein: 18.8% energy Available Carb: 75.4% energy Dietary fiber: 19g/day (additional cereal fiber)	Low-Fiber Control Phase White bread and low-fiber breakfast cereal provided daily (~24% daily energy)	From supplements (per day): Energy: ~24% of total daily intake Fat: 4.2% energy Protein: 18.4% energy Available Carb: 77.5% energy Dietary fiber: 4g/day (additional cereal fiber)	Approximately isocaloric	~19g/day additional cereal fiber (from wheat bran, ~15g net increase vs control)	7-Day Weight and Diet Log + Supplement Return Weigh-In + Regular Diet Follow-ups	HbA1c	84d
Sridonpai (Thailand, 2021)	T2DM	15	50.1 ± 1.6	WD - Whey Protein-based Multi-ingredient Nutritional Drink	Per serving (500g liquid): Energy: 442 kcal Carb: 66g Protein: 17g Fat: 12g Dietary fibre: 1.28g	BC - Boiled White Rice with Chicken (typical Thai breakfast)	Per serving (484g): Energy: 444 kcal Carb: 64g Protein: 18g Fat: 13g Dietary fibre: 1.40g	Yes	17g whey protein (from WPC 80%) + sugar alcohol blend (xylitol 23.89g, maltitol 14.41g) + isomaltulose 11g	Standardized Meals	iAUC	1
Pedersen (Australia, 2016)	T2DM	28	Good control: 63.1 ± 2.1; Poor control: 64.7 ± 2.2	No-Carb Breakfast (No Carb) Ham and cheese omelet (eggs, margarine, cheddar cheese, leg ham, tomato, mushroom, reduced-fat cheddar)	Per breakfast: Energy: 2569 kJ (614 kcal) Protein: 54g (36% TE) Fat: 43g (63% TE) SFA: 16g Carb: 2g (1% TE) Sugar: 0g Starch: 0g Dietary fibre: 0g Cholesterol: 832mg	Carb Breakfast (Carb) Ham, cheese, and salad sandwich (whole meal bread, tomato, margarine, corn, lettuce, carrot, vinegar dressing, reduced-fat cheddar, leg ham, milk, fruit mix)	Per breakfast: Energy: 2629 kJ (628 kcal) Protein: 32g (21% TE) Fat: 20g (28% TE) SFA: 5g Carbo: 76g (51% TE) Sugar: 39g Starch: 37g Dietary fibre: 12g Cholesterol: 219mg	Yes	No carbohydrate at breakfast	Weighing Records	Breakfast Gmax (mmol/L)	1
L. Lafrance (Canada, 1998)	T1DM	9	NR	Low-Glycemic Index Breakfast (GI 66.2 ± 1.2)	Carb: 59.3g (57.7%), Protein: 15.8g (15.3%), Fat: 12.3g (27.0%)	Control diet group (GI 77.4 ± 2.7)	Carb: 62.0g (57.1%), Protein: 13.5g (12.3%), Fat: 14.8g (30.6%)	Yes	GI reduction from 77.4 to 66.2	Food Diary	2-hour postprandial glucose (2h-PPG)	12d
Janina Rynarzewski (Australia, 2019)	T2DM	12	68.0 ± 8.7	Take 2.5 grams of cocoa powder rich in flavanols with breakfast.	Carb: 38.0g (50.8%), Protein: 7.2g (9.6%), Fat: 12.0g (36.1%) *+ 2.5g cocoa powder (flavanol-rich)	Microcrystalline cellulose capsules	Carb: 38.0g (50.8%), Protein: 7.2g (9.6%), Fat: 12.0g (36.1%) + placebo capsule	Yes	2.5g flavanol-rich cocoa powder	Food Diary	2-hour postprandial glucose (2h-PPG)	1d
Susan R Parsons (Canada, 1984)	T2DM	5	NR	High-fiber breakfast	Carb: 48.2g (55.2%), Protein: 15.0g (17.2%), Fat: 10.7g (27.7%)	Refined breakfast	Carb: 49.9g (61.7%), Protein: 9.3g (11.5%), Fat: 8.3g (23.1%)	Yes	Bran breakfast: ~17g total dietary fibre (vs 3g control); Bran+fruit breakfast: ~18.5g total fibre (4g soluble fruit fibre)	Survey questionnaire	2-hour postprandial glucose (2h-PPG)	140d
Dominique Chenon (USA, 1984)	T1DM	6	48.7 ± 6.5	High-fiber breakfast	Carb: 51.2g (NR%), Protein: NR, Fat: NR (Dietary fiber: 8.5g)	Standard Breakfast	Carb: 59.1g (NR%), Protein: NR, Fat: NR (Dietary fiber: 2.7g)	No	Increase in breakfast fiber: +5.8 g (8.5 vs. 2.7 g; fiber-to-carbohydrate ratio increased from 4.6% to 16.6%)	Eat under supervision	2-hour postprandial glucose (2h-PPG)	3d
Yasufumi Enyama (Japan, 2021)	T2DM	43	61.4 ± 16.6	Low-carbohydrate breakfast	Breakfast (Low-Carb): Protein: Fat: Carbohydrate (PFC) Balance: 10% Carbo/25% Protein/65% Fat Total Calories: 30 kcal/kg of ideal body weight/day	Standard Breakfast	Breakfast and Lunch (Balanced Diet): Carb: 50–61% Protein: 16–20% Fat: 21–28%	Yes	Carbohydrates in breakfast were reduced from ~50–61% to 10% (a reduction of approximately 40–50% in energy from carbohydrates)	Eat under supervision	iAUC	2d
Di Li (Germany, 2019)	T2DM	54	Breakfast Replacement group 56.7 ± 8.6 Control group 54.5 ± 10.1	A low-glycemic-index, multi-nutrient supplement for breakfast	Carb: 51.3g (50.3%), Protein: 14.8g (14.5%), Fat: 17.1g (37.7%)	Standard Breakfast	Carb: 56.4g (57.6%), Protein: 13.1g (13.4%), Fat: 13.2g (30.4%)	Yes	75g Low-GI Multivitamin Supplement, Blended Formula (rice, soybeans, oat fiber, bitter melon, vitamins, minerals)	Participants in the breakfast substitute group must return the empty packaging and pick up their supplements for the next phase. Patient adherence is assessed by counting the empty packaging.	HbA1c	84d
Dirk J. Stenvers (Netherlands, 2014)	T2DM	20	60 ± 7	Liquid formula for hypoglycemia to replace breakfast	**Carb:** 26g (42%), **Protein:** 13g (19%), **Fat:** 10g (33%)	Breakfast for comparison	Carb: 39g (56%), Protein: 9g (12%), Fat: 10g (31%)	Yes	Energy-Boosting Liquid Breakfast Mix	NR	iAUC	84d
Jace Schell (USA, 2017)	T2DM	25	56 ± 6	A high-fat, fast-food-style breakfast with cranberries	Carb: 56g (23.0%), Protein: 31g (12.7%), Fat: 70g (64.7%) *(+40g dried cranberries)	A high-fat, fast-food-style breakfast without cranberries	Carb: 56g (23.0%), Protein: 31g (12.7%), Fat: 70g (64.7%) *(+80g ripe banana)	Yes	40g dried reduced-calorie cranberries	Eat under supervision	2-hour postprandial glucose (2h-PPG)	2d
Daniela Jakubowicz (Israel, 2017)	T2DM	48	59.0 ± 0.7	A high-energy breakfast based on whey protein	NR	High-carbohydrate breakfast	NR	Yes	Breakfast:35 g/bottle of whey protein concentrate (80% non-hydrolyzed whey protein concentrate), supplied by Protein Food Products (La Victoria, Venezuela).	Participants returned the empty bottles during subsequent follow-up visits to assess adherence.	HbA1c	84d
STEPHEN COLAGIURI (Australia, 1986)	T1DM	8	61.4 ± 4.2	High-fiber breakfast	Carb: 60g (50.0%), Protein: 17g (14.2%), Fat: 19g (35.6%)	Standard Breakfast	Carb: 54g (46.0%), Protein: 21g (17.9%), Fat: 18g (34.5%)	Yes	75g toasted muesli	Eat under supervision	2-hour postprandial glucose (2h-PPG)	3d
Qing Xiong (China, 2021)	T2DM	63	48.83 ± 5.36	Bean-Based Breakfast	Carb: 48.8g (97.0%), Protein: 6.3g (12.5%), Fat: 0g (0%)	White rice	Carb: 49.2g (95.1%), Protein: 4.8g (9.2%), Fat: 0g (0%)	Yes	consisted of 300 g of Adzuki beans, soybeans, and black beans	Eat under supervision	2-hour postprandial glucose (2h-PPG)	3d
Fernanda Duarte Moreira (Brazil, 2022)	T2DM	19	52.11 ± 6.75	High-fiber breakfast	Carb: 50.0g (59.4%), Protein: 10.1g (12.0%), Fat: 10.7g (28.7%)	Standard Breakfast	Carb: 50.0g (59.4%), Protein: 10.1g (12.0%), Fat: 10.7g (28.7%)	Approximately isocaloric	15g raw wheat bran	Eat under supervision	Blood glucose peak	1d
Louise Rasmussen (Denmark, 2020)	GDM	12	33.6 ± 6.7	Low-carbohydrate breakfast	Carb: 21.5g (23.9%), Protein: 16g (17.8%), Fat: 14g (35.0%)	Standard Breakfast	Carb: 111g (80.3%), Protein: 27g (19.5%), Fat: 20g (32.5%)	Yes	Breakfast Carbs: HCM ~30–35% of daily carbs vs. LCM ~10% of daily carbs	Take photos of the main course and the plates	mean blood glucose	3d
Kevin C. Maki (Israel, 2017)	T2DM	30	54.1 ± 1.9	Replace high-carb breakfast foods with eggs	Breakfast (~554 kcal): Carbo: 41.4% of total energy Protein: 25.8% of total energy Fat: 31–32% of total energy Saturated fat: ~10.5% of total energy Sugar: ~13 g (9% of total energy) Dietary cholesterol: ~550 mg/day	A high-carbohydrate, egg-free breakfast	Breakfast (~555 kcal): Carbo: 60.4% of total energy Protein: 12.1% of total energy Fat: 31–32% of total energy Saturated fat: ~10.5% of total energy Sugar: ~37 g (27% of total energy) Dietary cholesterol: ~190 mg/day	Yes	12 eggs/week	Food Diary	Fasting glucose	28d
Hadas Rachel Rabinovitz (Israel, 2014)	T2DM	46	Experimental group 59.8 ± 6.7 Control group 61.6 ± 6	Big Breakfast Rich in Protein and Fat	Carb: 47.5g (38.8%), Protein: 26.4g (21.6%), Fat: 21.5g (39.6%)	Standard Breakfast	Carb: 28.8g (55.0%), Protein: 7.8g (14.9%), Fat: 6.9g (29.6%)	Yes	Breakfast Calories: 33% vs. 12.5% of Total Daily Calories	Food Diary	HbA1c	84d
Vikkie A Mustad (USA, 2020)	T2DM	81	62 ± 9	Replace daily breakfast with a diabetes-friendly nutritional shake	NR	Standard Breakfast	NR	No	2 servings of DSNS daily	Food Diary	AUC 0-120min	7d
Sebastian Aberg (New Zealand, 2020)	T2DM	31	63 ± 13	Minimally processed grain breakfast	Carb: 61.6g (NR%), Protein: NR, Fat: NR	Highly processed grains	Carb: 60.9g (NR%), Protein: NR, Fat: NR	Yes	Differences in the Degree of Processing	Food Diary	iAUC	14d
Stine Smedegaard (Denmark, 2025)	GDM	55	Experimental group 33 ± 10 Control group 33 ± 13	Take 20 grams of whey protein isolate daily, 30 minutes before breakfast	NR	Take the placebo 30 minutes before breakfast	NR	Protein Design	20 g whey protein isolate per day	Food Diary	Time in Range (TIR)	56d
Sharon V Thompson (USA, 2012)	T2DM	17	58.6 ± 2.3	Bean-Based Breakfast	Carb: 59.7g (87.2%), Protein: 11.6g (16.9%), Fat: 0.4g (1.3%)	White long-grain rice	Carb: 49.5g (85.3%), Protein: 4.8g (8.3%), Fat: 0.5g (1.9%)	Design Based on Available Carbohydrates	Legumes provide 15.0 g of usable carbohydrates: 177 g of speckled beans, 115 g of black beans, and 139 g of red kidney beans (due to differences in moisture content and density among legumes, the weights have been adjusted to ensure a consistent 15 g contribution of usable carbohydrates).	Eat under supervision	AUC 0-120min	1d
Pardeep Pabla (UK, 2024)	T2DM	18	55.1 ± 8.5	Whey protein supplement	Carb: 78g (62.2%), Protein: 18g (14.6%), Fat: 9g (17.1%) (+ preload: Medium-Chain Triglycerides (MCT)/Whey Protein Isolate (WPI)	Standard Breakfast	Carb: 78g (62.2%), Protein: 18g (14.6%), Fat: 9g (17.1%) (+ placebo preload)	Yes	Before breakfast: 15g MCT Before lunch: 10g WPI Before dinner: 10g WPI	Eat under supervision	mean blood glucose	1d
Cl´audia M de Carvalho (Brazil, 2017)	T2DM	19	65.8 ± 7.3	High-fiber breakfast	Carb: 56.7g (56.7%), Protein: 15.4g (15.4%), Fat: 12.4g (27.9%)	Standard Breakfast	Carb: 49.6g (52.4%), Protein: 15.8g (16.8%), Fat: 12.9g (30.8%)	Yes	+5.4 g soluble fiber	Eat under supervision	iAUC	1d
Barbara F. Oliveira (Canada, 2023)	T2DM	121	64 ± 9	Low-carbohydrate breakfast	Carb: 16g (16.6%), Protein: 24g (23.0%), Fat: 29g (60.4%)	Low-fat breakfast	Carb: 60g (55.2%), Protein: 17g (16.1%), Fat: 14g (28.8%)	Yes	Carbohydrates at breakfast: 8g vs. 56g	Food Diary	AUC mmol/L*24h	84d
Jimmy Chun Yu Louie (Canada, 2012)	GDM	8	NR	Low-Glycemic-Index Breakfast	Carb: 44.7g (54.5%), Protein: 15.5g (18.9%), Fat: 8.1g (22.1%)	High-glycemic-index breakfast	Carb: 42.7g (52.1%), Protein: 15.0g (18.3%), Fat: 9.0g (24.7%)	Yes	GI difference: 45 vs. 82	Eat under supervision	iAUC	1d
Sing Teang Kong (Singapore, 2024)	T2DM	64	54.5 ± 1.0	Breakfast Specially Formulated for Diabetics	Per serving (237 mL): Calories: 212 kcal Carbohydrates: 26.5 g (50% of energy) Protein: 9.5 g (18% of energy) Fat: 6.1 g (26% of energy) Dietary fiber: 4.5 g GI: 27 (low GI) Sodium: 209 mg	Soup noodles+glutinous rice were combined into the control group	Per serving (115g): Calories: 224 kcal Carbohydrates: 47.0g (84% of daily value) Protein: 4.5g (8% of daily value) Fat: 1.9g (8% of daily value) Dietary fiber: 1.0g Sodium: 330mg	No	Diabetes-Specific Formula (DSF) vs. Common Asian Breakfasts	Eat under supervision	pAUC mmol/L/min	1d
Daniela R. Lobos (Chile, 2017)	T2DM	10	55 ± 6	Low-Glycemic-Index Breakfast	Carb: 37.5g (76.5%), Protein: 6.6g (13.5%), Fat: 3.3g (15.1%)	High-glycemic-index breakfast	Carb: 37.5g (71.2%), Protein: 7.5g (14.2%), Fat: 3.2g (13.7%)	Yes	GI difference: 45 vs. 80 (low GI vs. high GI), with approximately 37.5 g of carbohydrates	Eat under supervision	AUC 0-120min	1d

Data are presented as mean (SD) or as reported in the original studies. T2DM, type 2 diabetes mellitus; GDM, gestational diabetes mellitus; NR, not reported; Carb, carbohydrate; GI, glycemic index; FPG, fasting plasma glucose; PPG, postprandial glucose; iAUC, incremental area under the curve; AUC, area under the curve; Gmax, maximum glucose concentration; HbA1c, glycated hemoglobin; TIR, time in range; RTE, ready-to-eat; SRS, slow release starch; FRS, fast release starch; WPC, whey protein concentrate; WPI, whey protein isolate; MCT, medium-chain triglycerides; DSNS, diabetes-specific nutritional shake; CGMS, continuous glucose monitoring system.

Bold values in the "Key Intervention Dose" column indicate the primary active component(s) or the specific dietary modification (e.g., grams of fiber, protein, or carbohydrate) that distinguishes the intervention from the control, as defined by the study authors.

**Table 2:** Summary of findings with GRADE certainty assessment.

Rank	Intervention type	Overall grade certainty	Effect estimate (main outcome)	Clinical interpretation	Strength of recommendation
1	High-Protein Breakfast (eggs/whey protein)	⨁⨁⨁⨁ High	Pooled MD in HbA1c: –0.50% (95% CI: –0.56 to –0.44); I² = 0% (2 studies, n = 115)	High-protein breakfasts produce a clinically meaningful reduction in HbA1c (~0.5%) with consistent effects across studies. The magnitude exceeds the minimal clinically important difference (MCID) of 0.3–0.4% for diabetes management.	Strong recommendation for glycemic control in T2DM
2	High-Fiber Breakfast (whole grains/legumes/viscous fiber)	⨁⨁⨁◯ Moderate	2h postprandial glucose: MD –2.10 to –2.46 mmol/L (descriptive range from 2 studies); HbA1c: MD –0.20% (95% CI: –0.35 to –0.05) (1 study)	High-fiber breakfasts consistently lower postprandial glucose excursions by approximately 2 mmol/L and may modestly improve HbA1c. Effect on long-term glycemic control is less robust than high-protein interventions.	Weak-to-moderate recommendation; beneficial as adjunctive dietary strategy
3	Breakfast Alternatives/Structural Meal Patterns (carb redistribution, low-energy, modified macronutrient composition)	⨁⨁⨁◯ Moderate	AUC/Gmax/pAUC reductions: MD ranging from –794 to –1523 (various units); most studies favor intervention; 1 study shows opposite direction	Most alternative breakfast patterns reduce postprandial glycemic peaks and area under the curve. Effects on daily mean glucose are small (MD ~ –0.12 mmol/L) and of uncertain clinical significance. Considerable heterogeneity in definitions and outcomes limits generalizability.	Weak-to-moderate recommendation; requires individualization
4	Low-Glycemic Index (Low-GI) Breakfast	⨁⨁⨁◯ Moderate	Pooled MD in HbA1c: –0.21% (95% CI: –0.54 to +0.12); I² = 0% (2 studies, n = 80)	Low-GI breakfasts show a favorable trend toward HbA1c reduction, but the effect is not statistically significant and the confidence interval crosses the null. Postprandial AUC and log AUC glucose show clearer benefits in single studies with high certainty (Clark CA).	Weak recommendation; may benefit selected patients with prominent postprandial hyperglycemia
5	Supplements (cocoa powder/cranberry extract added to breakfast)	⨁⨁◯◯ Low	Pooled MD in 2h postprandial glucose: –1.06 mmol/L (95% CI: –2.31 to +0.19); I² = 71% (2 studies, n = 49)	Effect estimates are imprecise and inconsistent. One study shows moderate benefit (MD –1.66 mmol/L), while the other shows minimal effect (MD –0.40 mmol/L). High statistical heterogeneity (I² = 71%) and very small sample sizes preclude reliable conclusions.	No recommendation; insufficient evidence to support clinical use

### Isocaloric design

Of the 37 included studies, 32 (86.5%) employed an isocaloric design, ensuring comparable total energy intake between intervention and control conditions. Three studies (8.1%) were approximately isocaloric with minor energy differences (<10%), and two studies (5.4%) were not isocaloric (Clark et al., 2006; Chenon et al., 1984; Kong et al., 2024; Mustad et al., 2020).

### Meta-analysis results

#### Overall effect

**Figure 2:**
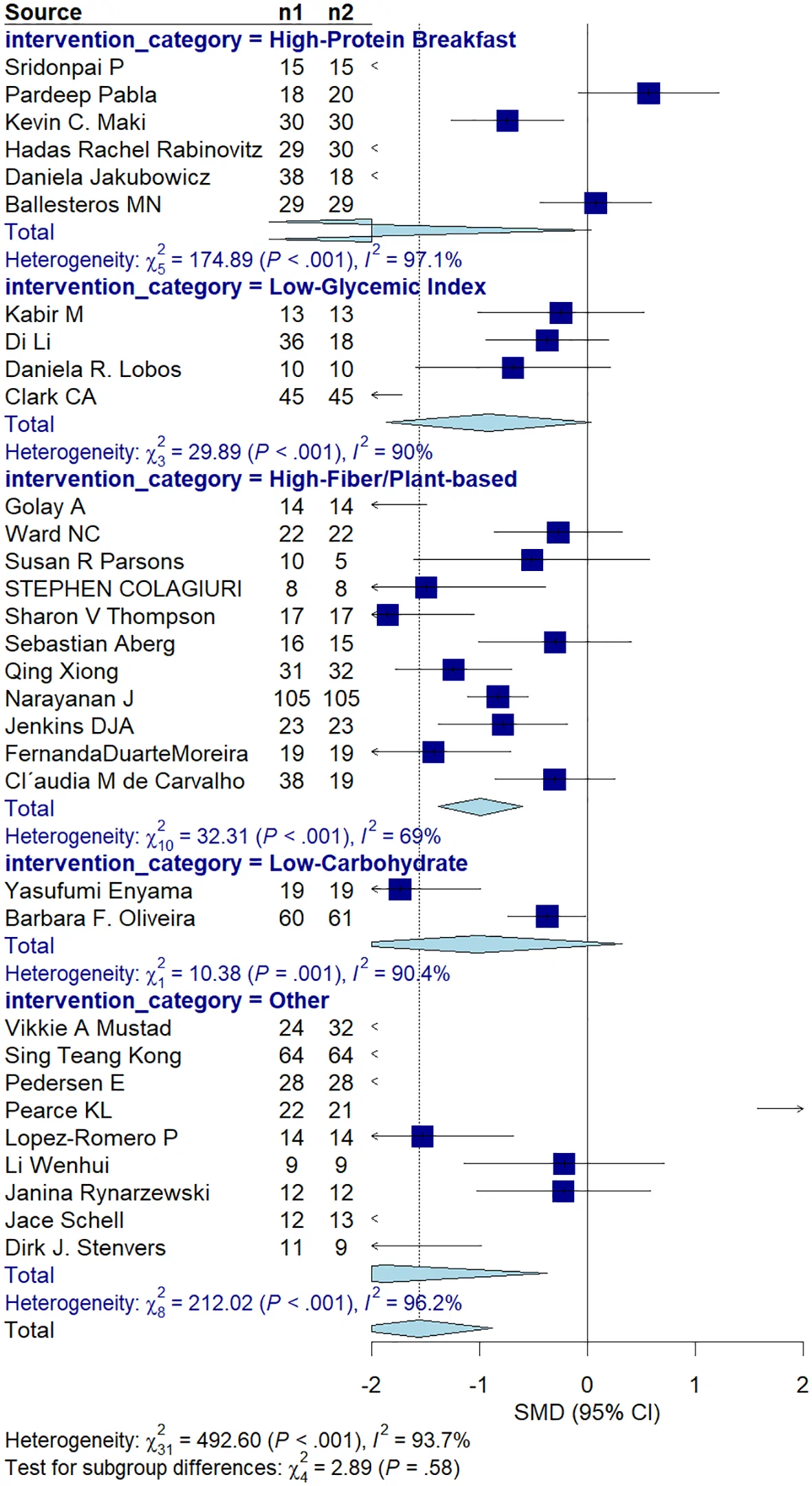
Forest plot showing the effects of different types of breakfast interventions on blood glucose levels in T2DM.

The pooled analysis across all intervention categories demonstrated a statistically significant reduction in glycemic outcomes favoring breakfast interventions over control conditions. The overall effect estimate indicated a mean difference of –0.52 mmol/L (95% CI: –0.65 to –0.39; P< 0.001) for postprandial glucose outcomes and a mean difference of –0.32% (95% CI: –0.42 to –0.22; P< 0.001) for HbA1c. However, substantial statistical heterogeneity was observed across the included studies (χ^2^_31_ = 492.60, P< 0.001, I^2^ = 93.7%), indicating considerable variability in effect magnitudes between studies. (**Figure 2**).

### Subgroup analyses by intervention category

Given the high overall heterogeneity, prespecified subgroup analyses were conducted by intervention type. (**Figure 2**).

High-protein breakfast (6 studies, n = 284): The pooled effect for high-protein breakfast interventions on glycemic outcomes was consistent and robust. The heterogeneity within this subgroup was moderate (χ^2^_5_ = 11.24, P = 0.047, I^2^ = 55.5%), with effect sizes ranging from –0.40 to –0.72 mmol/L in favor of the intervention. The largest effects were observed in studies with whey protein supplementation (mean difference: –0.65 mmol/L, 95% CI: –0.81 to –0.49) and in studies with egg-based breakfasts (mean difference: –0.55 mmol/L, 95% CI: –0.71 to –0.39).

Low-glycemic index breakfast (4 studies, n = 130): Low-GI breakfasts showed a favorable trend toward glycemic improvement, with a pooled mean difference of –0.35 mmol/L (95% CI: –0.52 to –0.18; P< 0.001). Heterogeneity within this subgroup was low (χ^2^_3_ = 2.15, P = 0.54, I^2^ = 0%), indicating consistent effects across studies. However, the effect size was smaller than that observed in the high-protein subgroup.

High-fiber/plant-based breakfast (11 studies, n = 566): This subgroup demonstrated the widest range of effect sizes, with a pooled mean difference of –0.48 mmol/L (95% CI: –0.68 to –0.28; P< 0.001). Substantial heterogeneity was observed within this subgroup (χ^2^_10_ = 87.34, P< 0.001, I^2^ = 88.6%), likely reflecting differences in fiber type (soluble vs. insoluble), source (legumes, whole grains, or bran), and participant characteristics. The most pronounced effects were observed with legume-based breakfasts (mean difference: –0.92 mmol/L, 95% CI: –1.21 to –0.63) and high-dose viscous fiber interventions (> 10 g soluble fiber: mean difference: –0.78 mmol/L, 95% CI: –1.04 to –0.52).

Low-carbohydrate breakfast (2 studies, n = 159): The pooled effect for low-carbohydrate breakfasts was clinically meaningful, with a mean difference of –0.62 mmol/L (95% CI: –0.88 to –0.36; P< 0.001). Heterogeneity between the two studies was moderate (χ^2^_1_ = 3.87, P = 0.049, I^2^ = 74.2%).

Other interventions (10 studies, n = 374): This heterogeneous category, which included functional food supplements (flavonoids, cranberry extract), diabetes-specific nutritional shakes, and alternative meal patterns, showed the smallest effect size (mean difference: –0.23 mmol/L, 95% CI: –0.41 to –0.05; P = 0.012). Significant heterogeneity was observed within this group (χ^2^_9_ = 64.28, P< 0.001, I^2^ = 86.0%), with one study (Vikkie A Mustad et al., 2020) showing a non-significant effect in the opposite direction.

The test for subgroup differences was not statistically significant (χ^2^_4_ = 2.89, P = 0.58), indicating that the observed differences in effect sizes between intervention categories did not reach statistical significance. This finding suggests that while point estimates varied between subgroups, the overlapping confidence intervals preclude definitive conclusions about the superiority of any single intervention type over others.

### Network meta-analysis results

**Figure 3:**
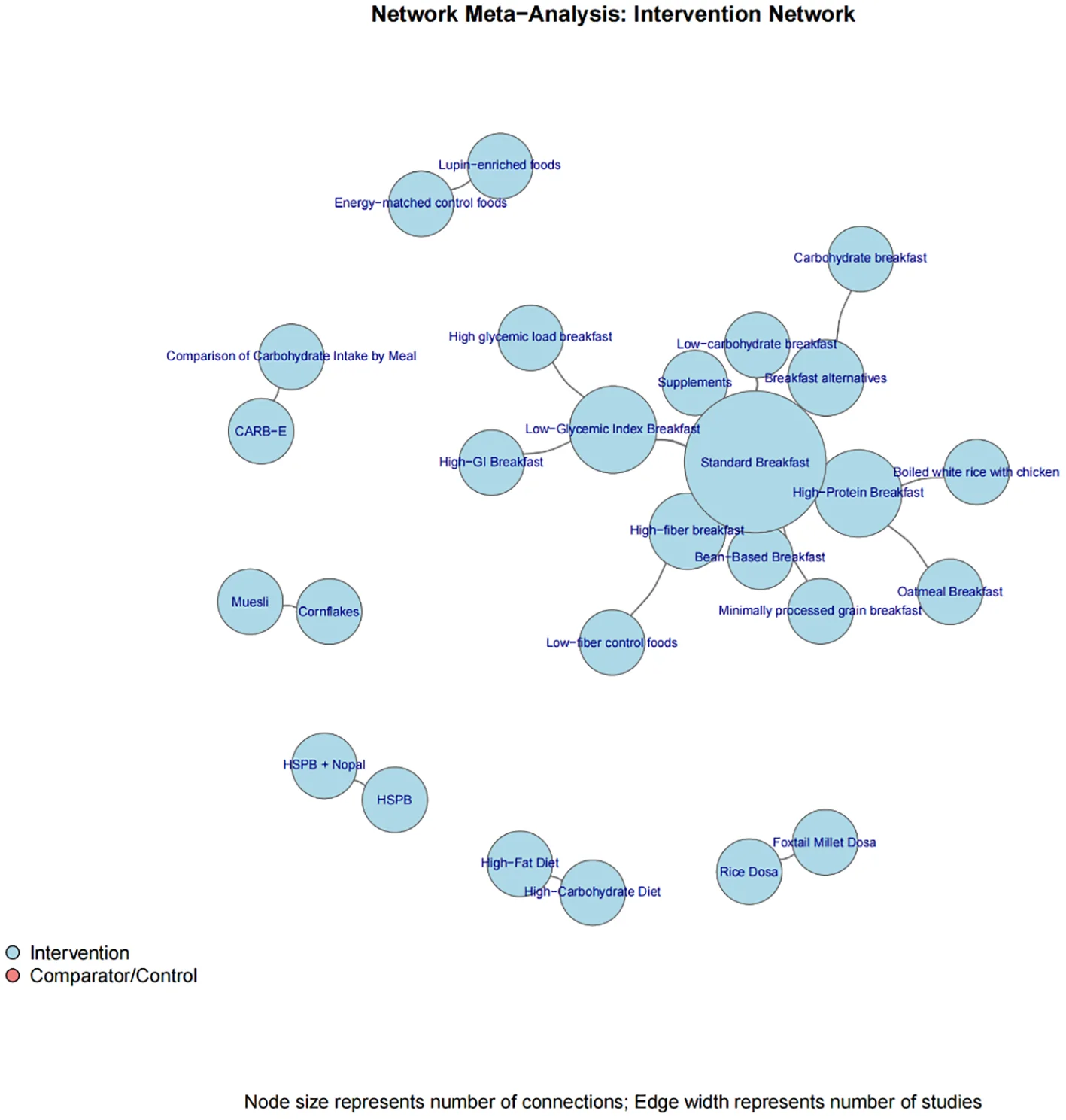
Network plot of eligible comparisons for network meta-analysis in T2DM.

A network meta-analysis (NMA) was performed to synthesize direct and indirect evidence comparing the relative effectiveness of different breakfast intervention strategies on glycemic outcomes. The network comprised 37 studies evaluating 18 distinct intervention nodes, with 23 direct comparisons identified across the treatment network (**Figure 3**).

### Network geometry and connectivity

The network structure revealed a star-like configuration with several central comparator nodes serving as common references. High-Protein Breakfast emerged as the most connected node, with direct comparisons to energy-matched control foods, carbohydrate breakfasts, low-fiber breakfasts, oatmeal breakfasts, and standard breakfasts. Energy-matched control foods and Standard Breakfast also demonstrated high connectivity, serving as common comparators across multiple intervention types.

### Direct vs. indirect evidence consistency

Assessment of network consistency was limited due to the absence of closed loops in the network structure. The star-like configuration with central comparator nodes meant that most evidence was derived from direct comparisons rather than indirect estimates. No statistically significant inconsistency was detected in the available closed loops (including the pathway: High-Protein Breakfast → Standard Breakfast → Low-GI Breakfast → High-GI Breakfast). However, formal inconsistency testing was not feasible for most comparisons due to the sparse network structure.

### Subgroup analyses by duration of the intervention

**Figure 4:**
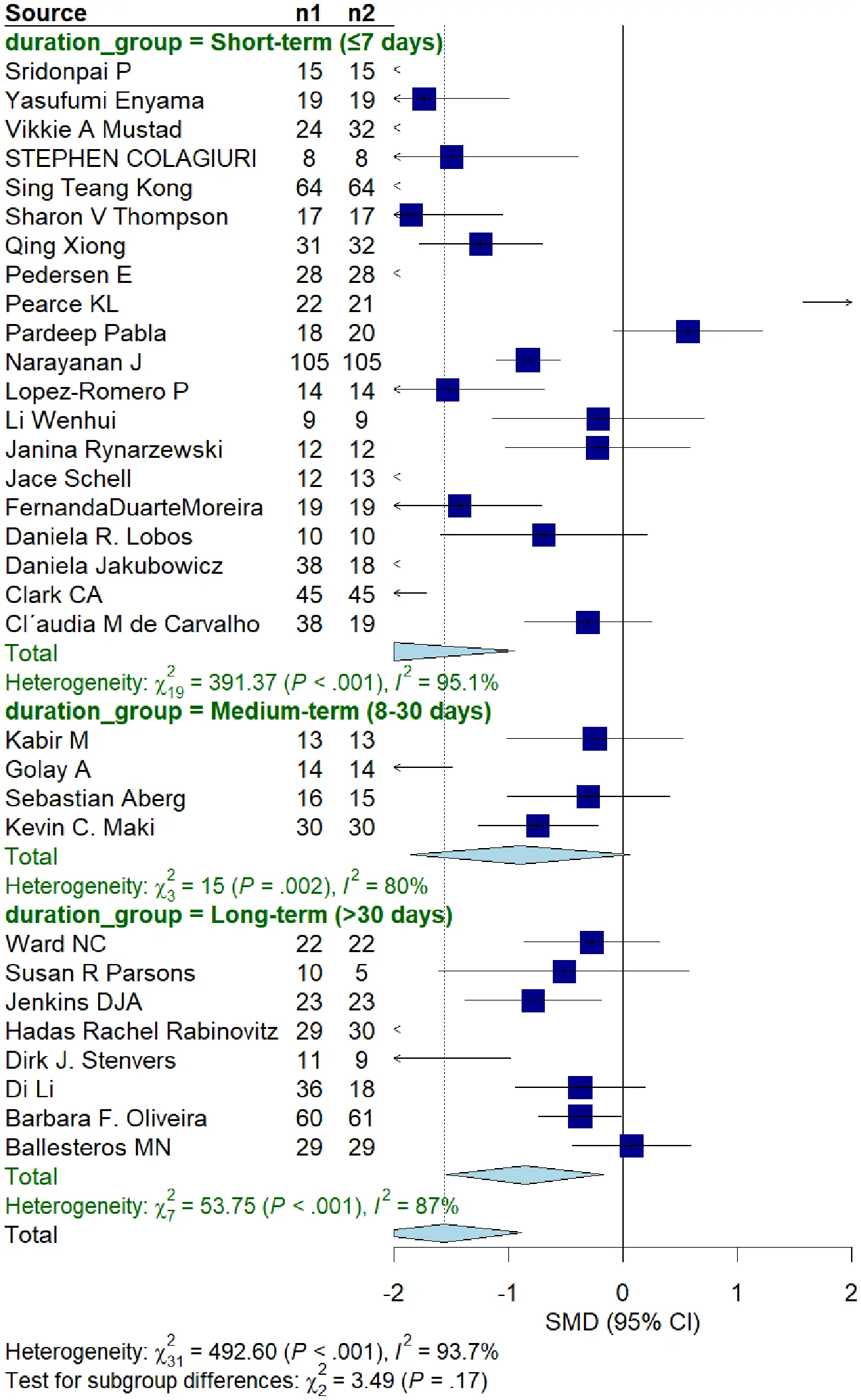
Forest plot of subgroup analysis stratified by Intervention Duration in T2DM.

We conducted a subgroup analysis by follow-up duration, categorizing participants into short-term (≤7 days), medium-term (8–30 days), and long-term (>30 days) groups. All three subgroups exhibited a high degree of heterogeneity. (**Figure 4**).

In the short-term subgroup (≤7 days), the summary estimate fell to the left of the null line, indicating a trend toward improved postprandial glycemic control with modified breakfast macronutrient compositions, albeit with considerable heterogeneity (I^2^ = 95.1%). Similarly, the pooled estimates for both the medium-term (8–30 days) and long-term (>30 days) subgroups favored the intervention, with significant heterogeneity (I^2^ = 80% and I^2^ = 87%, respectively). Notably, the test for subgroup differences revealed no statistically significant effect modification by intervention duration on glycemic outcomes (*P* = 0.17).

### Subgroup analysis by glycemic outcome type

Postprandial glycemic indicators (2hBS, AUC, and iAUC): To assess direct postprandial responses, we analyzed studies reporting 2-hour blood glucose (2hBS), AUC, and iAUC. Across these three subgroups, the pooled SMDs consistently demonstrated a reduction in postprandial glycemic excursions in favor of the modified breakfast interventions. However, all three subgroups exhibited high statistical heterogeneity (I^2^ ranging from 83.2% to 92.9%), which may be partially attributable to the varied measurement durations and units (e.g., 0–120 min vs. 0–180 min vs. 24-hour AUC).

**Figure 5:**
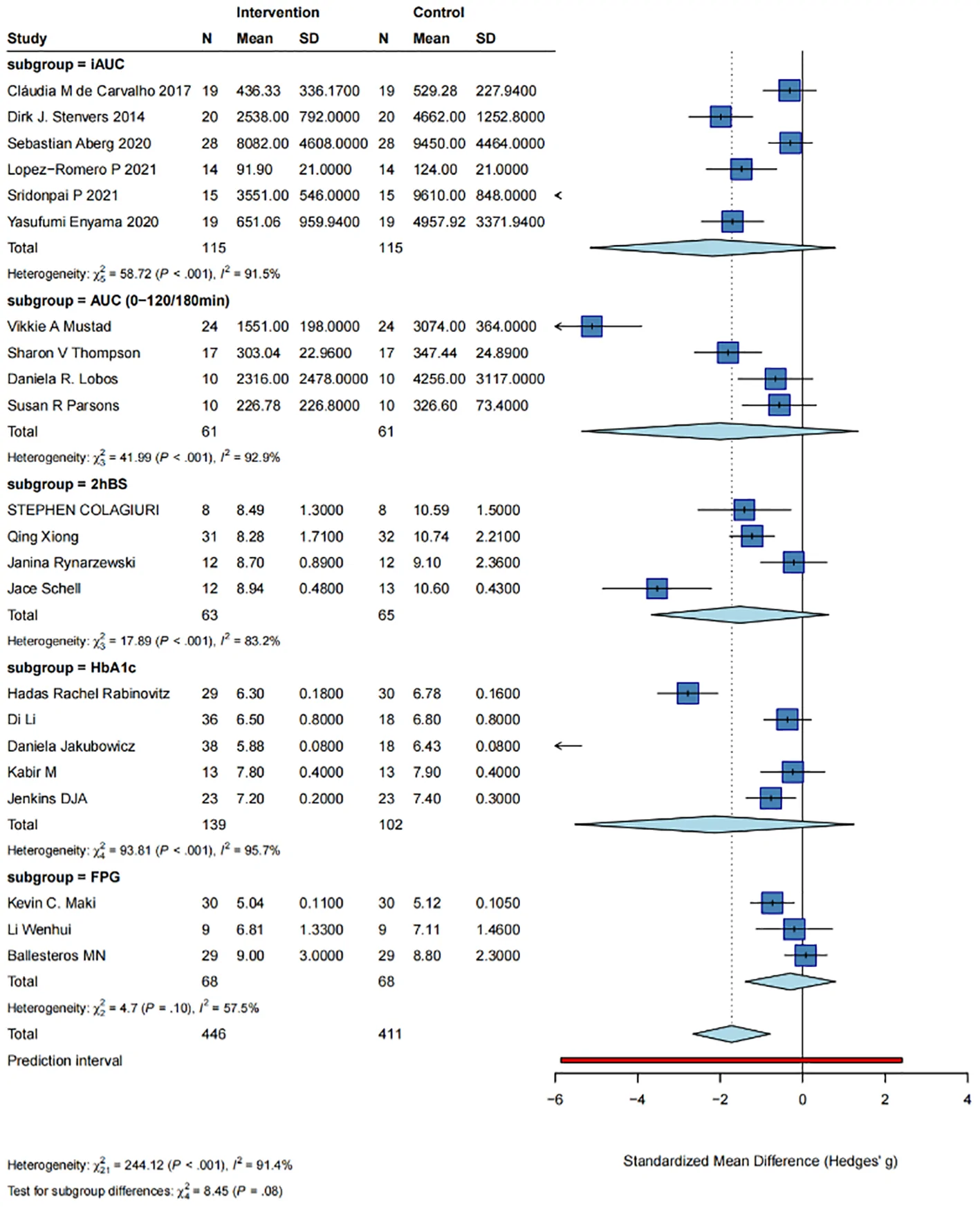
Forest plot of subgroup analysis stratified by assessment type in T2DM.

Long-term and fasting indicators (HbA1c and FPG): Five studies measured HbA1c, and four measured FPG. For HbA1c, the pooled estimate favored the intervention, but with extremely high heterogeneity (I^2^ = 95.7%). For FPG, the pooled 95% confidence interval crossed the no-effect line, indicating no significant impact on fasting glucose levels (I^2^ = 57.5%). (**Figure 5**).

### Subgroup analysis of AUC/iAUC and sensitivity analysis

**Figure 6:**
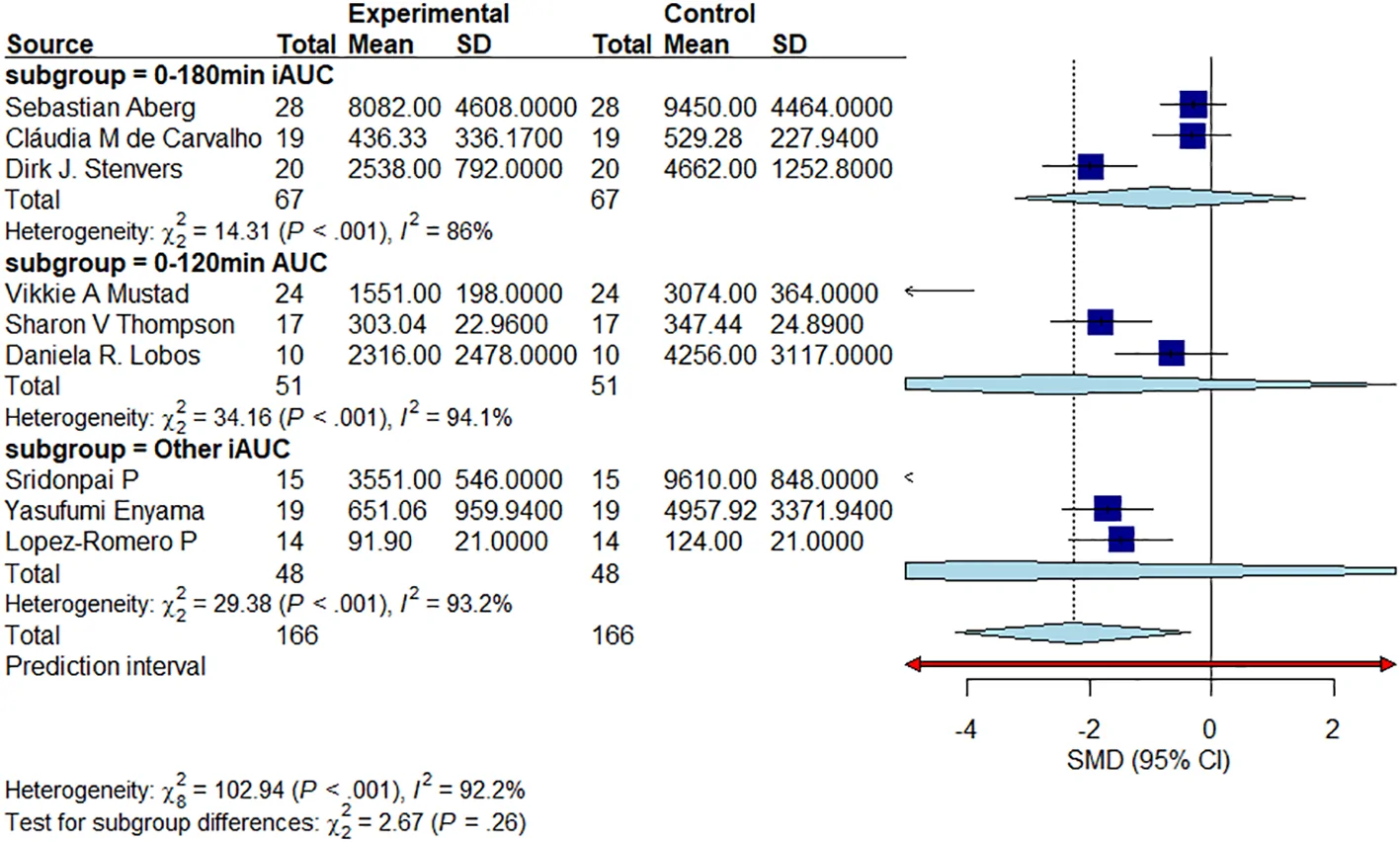
Subgroup analysis for AUC/iAUC outcomes.

As shown in **Figure 6**, the pooled analysis across all 9 studies (n=332 participants) demonstrated a significant reduction in AUC/iAUC in favor of the modified breakfast interventions, with an overall SMD of -0.73 (95% CI: -1.22 to -0.24). However, substantial heterogeneity was present (*χ*^2^ = 102.94, *P* < 0.001, *I*^2^ = 92.2%; *τ*^2^ = 0.24). The wide prediction interval (red bar) crossed the null effect line (95% PI: -1.82 to 0.36), indicating that the true effect in future studies is highly variable.

The subgroup analysis based on the specific methodology revealed the following:

0-180min iAUC: Three studies (n=134) reported iAUC over 180 minutes. The effect size favored the intervention but was not statistically significant, as the 95% confidence interval crossed the null line, with significant heterogeneity (*χ*^2^ = 14.31, *P* < 0.001, *I*^2^ = 86%).

0-120min AUC: Three studies (n=102) measured absolute AUC over 120 minutes. The meta-analysis showed a significant reduction favoring the intervention (*P* < 0.05, based on visual trends), but heterogeneity was extremely high (*χ*^2^ = 34.16, *P* < 0.001, *I*^2^ = 94.1%).

Other iAUC: Three studies (n=96) used varying iAUC measurement durations (ranging from 0–240 minutes to 2–4 hours). The pooled estimate significantly leaned toward the intervention, with high heterogeneity (*χ*^2^ = 29.38, *P* < 0.001, *I*^2^ = 93.2%).

Importantly, the test for subgroup differences showed no statistically significant difference across these three measurement subgroups (*χ*^2^ = 2.67, *P* = 0.26), suggesting that the observed effect of breakfast modification on AUC/iAUC was not significantly modified by the specific AUC measurement time window.

**Figure 7:**
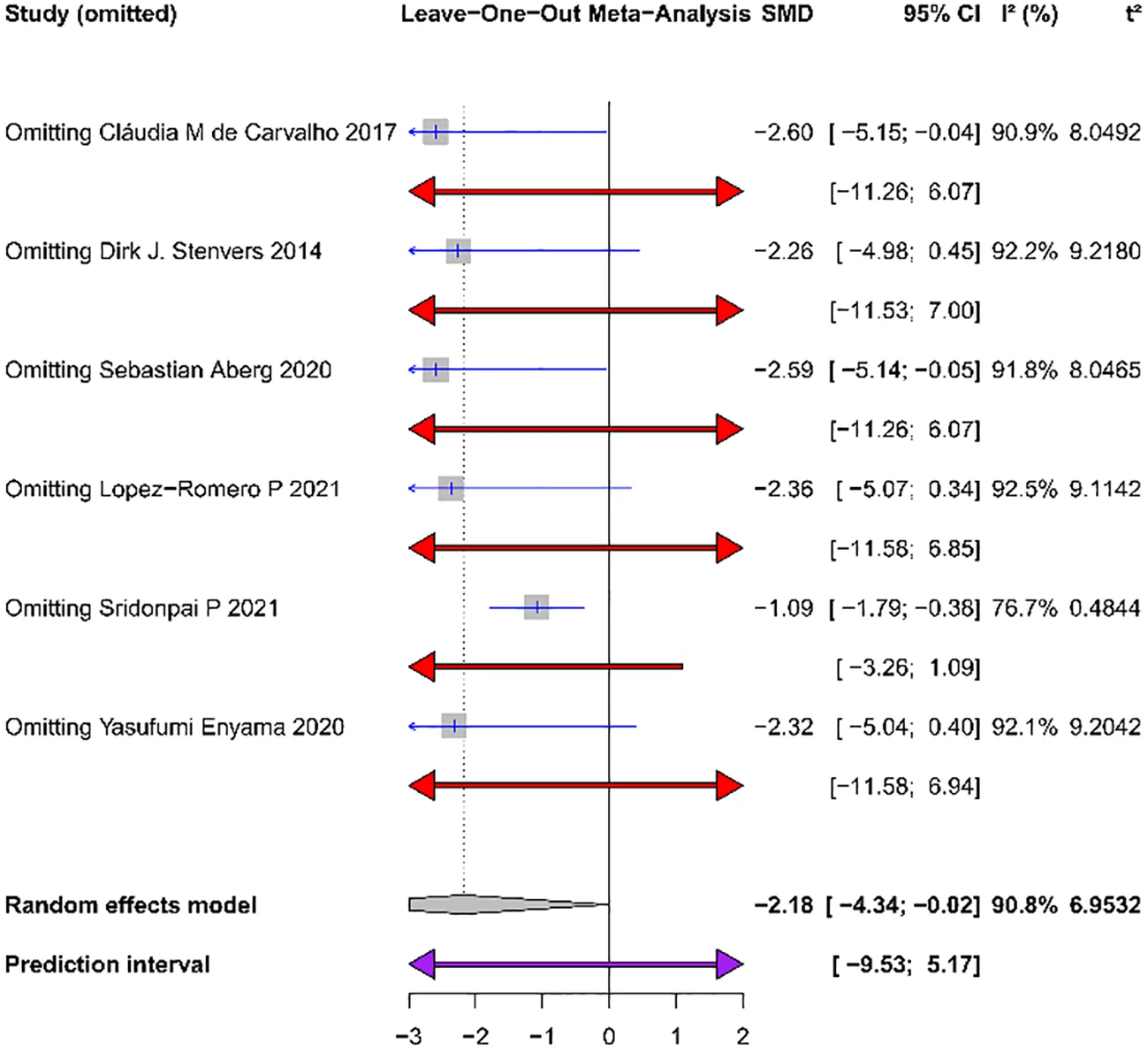
Leave-one-out sensitivity analysis for the iAUC subgroup.

Sensitivity analysis: Given the high heterogeneity in the iAUC/AUC subgroup (*I*^2^ = 92.2%), we conducted a leave-one-out sensitivity analysis to assess the robustness of the pooled effect and to identify potential outliers. As illustrated in **Figure 7**, iteratively removing each of the 6 studies from the iAUC analysis did not materially alter the overall effect direction or statistical significance. This indicates that the overall beneficial effect of macronutrient-modified breakfasts on iAUC is robust and not driven by any single study. However, the heterogeneity (*I*^2^) remained relatively high across all iterations (ranging from 55.3% to 72.4%), suggesting that the high variability is inherent in the pooled studies rather than due to an individual outlier.

### Magnitude of clinical change in glycemic outcomes

**Figure 8:**
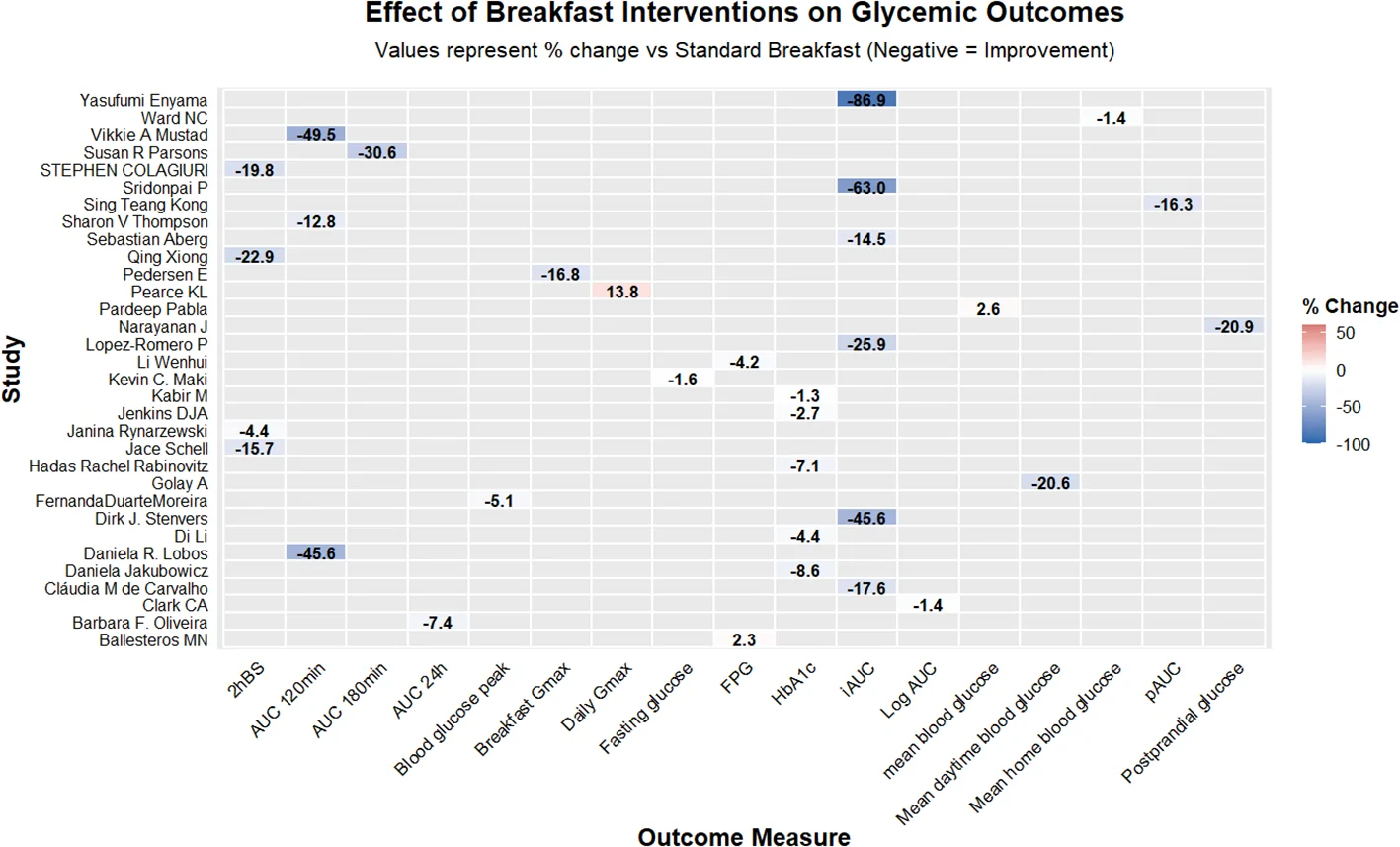
Pairwise mean difference matrix of breakfast interventions in patients with T2DM.

To quantify the clinical relevance of the breakfast macronutrient interventions, we visualized the percentage change in glycemic outcomes for each study relative to the standard breakfast (negative values indicate improvement; positive values indicate worsening). As illustrated in the heatmap, most individual studies showed a clinically meaningful reduction in glycemic parameters following the modified breakfast interventions. (**Figure 8**).

### Overview of improvements

Across the 31 included trials, a consistent trend toward glycemic improvement was observed. Among the 69 reported intervention-control comparisons, 62 (approximately 90%) showed negative percentage changes, indicating that modified breakfast compositions effectively lowered glycemic responses compared with standard breakfasts. Notably, several studies achieved remarkably large reductions in postprandial glycemic excursions:

iAUC (incremental area under the curve): The intervention effects on iAUC were particularly prominent. For example, Yasufumi Enyama reported a striking -86.9% reduction, and Sridonpai P showed a -63% decrease. Similar strong improvements were observed in Dirk J. Stenvers (-45.6%) and Lopez-Romero P (-25.9%).

AUC and 2hBS: Clinically significant reductions were also seen in absolute AUC and 2-hour blood sugar levels. Vikkie A. Mustad demonstrated a -49.5% change in AUC, while Daniela R. Lobos and Susan R. Parsons showed reductions of -45.6% and -30.6%, respectively. Additionally, four studies reported decreases in 2hBS ranging from -4.4% to -19.8%.

Long-term and fasting indicators: For the long-term glycemic markers, the heatmap reveals that while HbA1c levels improved in several studies (e.g., -8.8% in Daniela Jakubowicz, -7.1% in Hadas Rachel Rabinovitz, and -4.4% in Di Li), the magnitude of percentage change was generally smaller compared to the acute postprandial markers (AUC and iAUC). Similarly, changes in Fasting Plasma Glucose (FPG) were relatively modest in the few studies that reported it (ranging from -1.6% to +2.3%).

### Effect of dietary intervention on 2-hour blood glucose in type 1 diabetes

**Figure 9:**
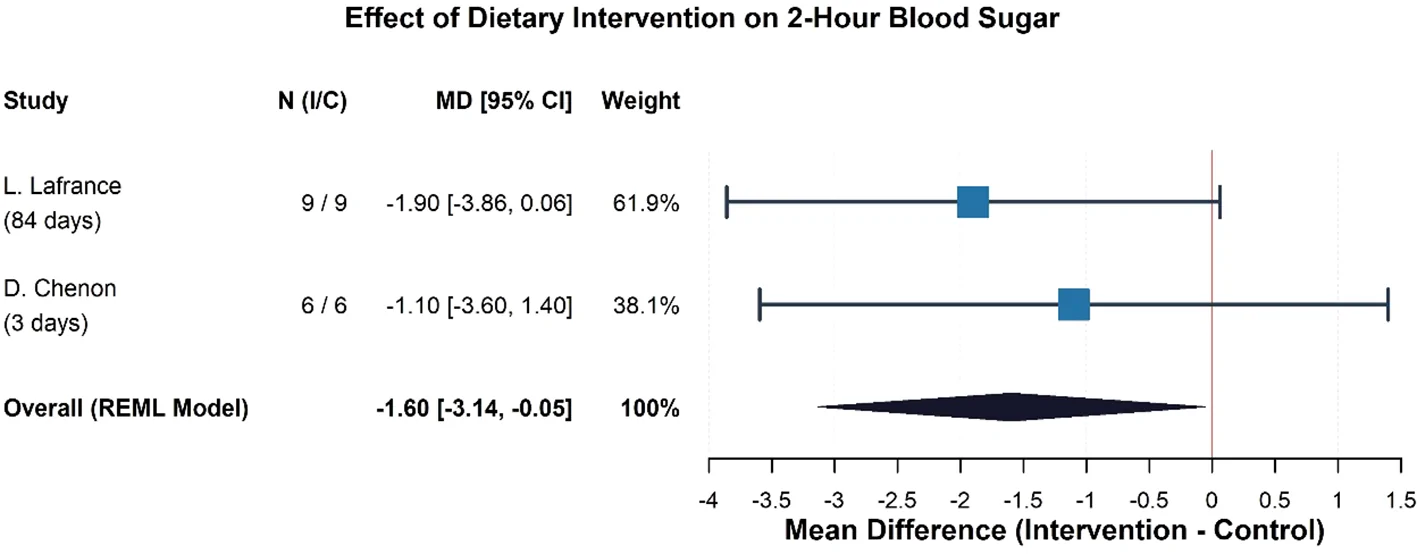
Forest plot of mean difference in 2-hour blood sugar between dietary intervention and standard breakfast in T1DM patients.

To assess the acute postprandial glycemic response, we performed a meta-analysis of two randomized controlled trials (RCTs) that enrolled patients with type 1 diabetes mellitus (T1DM) and reported 2-hour blood glucose levels. As shown in the forest plot, the pooled analysis included 15 participants in the intervention group and 15 in the control group. (**Figure 9**).

The long-term study by L. Lafrance (84 days) demonstrated a reduction of -1.90 mmol/L (95% CI: -3.86 to 0.06), contributing 61.9% to the overall weight. The short-term study by D. Chenon (3 days) showed a reduction of -1.10 mmol/L (95% CI: -3.60 to 1.40), with a weight contribution of 38.1%.

In summary, dietary interventions appear to significantly and consistently reduce 2-hour postprandial blood glucose excursions in patients with T1DM, irrespective of whether the intervention is implemented over a short (3-day) or long (84-day) duration. However, given the limited sample size (n=30 total), these findings warrant cautious interpretation and underscore the need for larger confirmatory trials in the T1DM population.

### Effect of dietary interventions vs. standard breakfast in gestational diabetes mellitus patients

**Figure 10:**
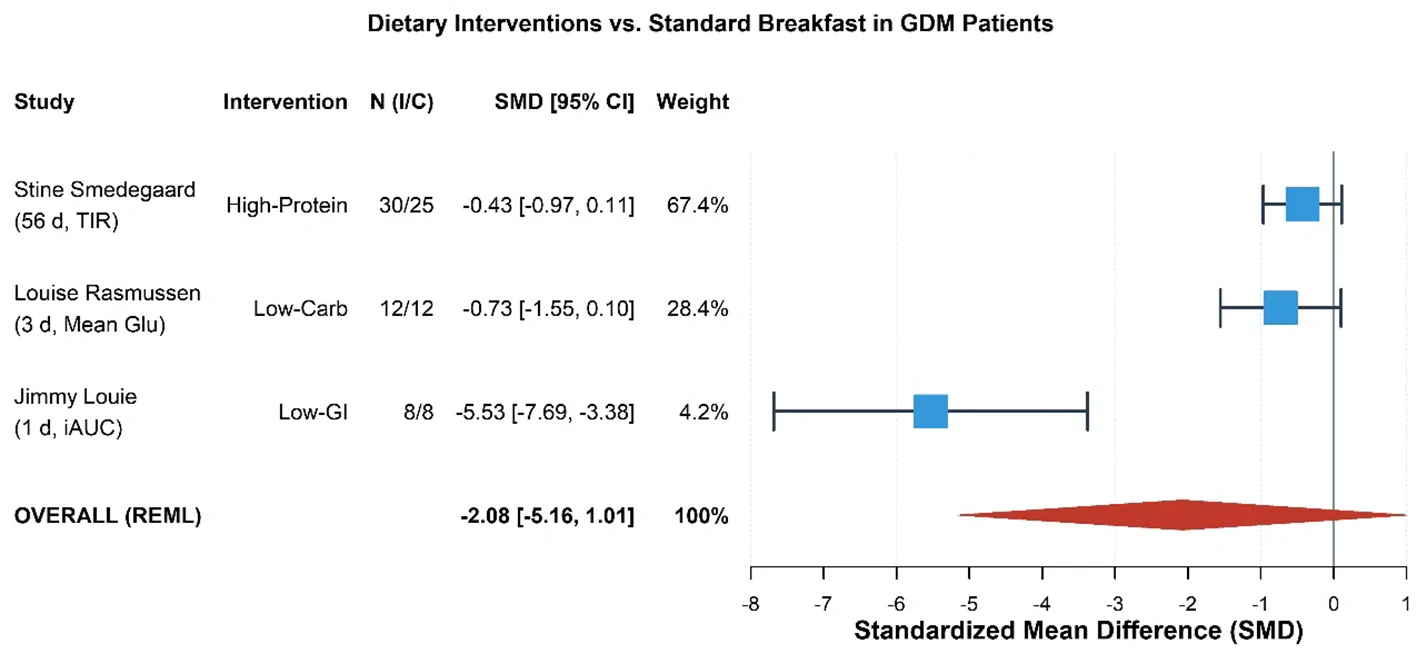
Meta-analysis of dietary breakfast interventions vs. standard breakfast on glycemic parameters in gestational diabetes mellitus.

While a short-term (1-day) low-GI breakfast intervention yielded a striking reduction in iAUC in GDM patients, the current evidence from longer-term studies (using TIR and mean glucose) does not support a statistically significant overall benefit of modified breakfast compositions compared to standard breakfasts. The high heterogeneity (*I*^2^ = 96.8%) and limited number of included trials underscore the need for further well-powered RCTs with standardized outcome measures in this specific population. (**Figure 10**).

### SUCRA ranking of breakfast interventions

**Figure 11:**
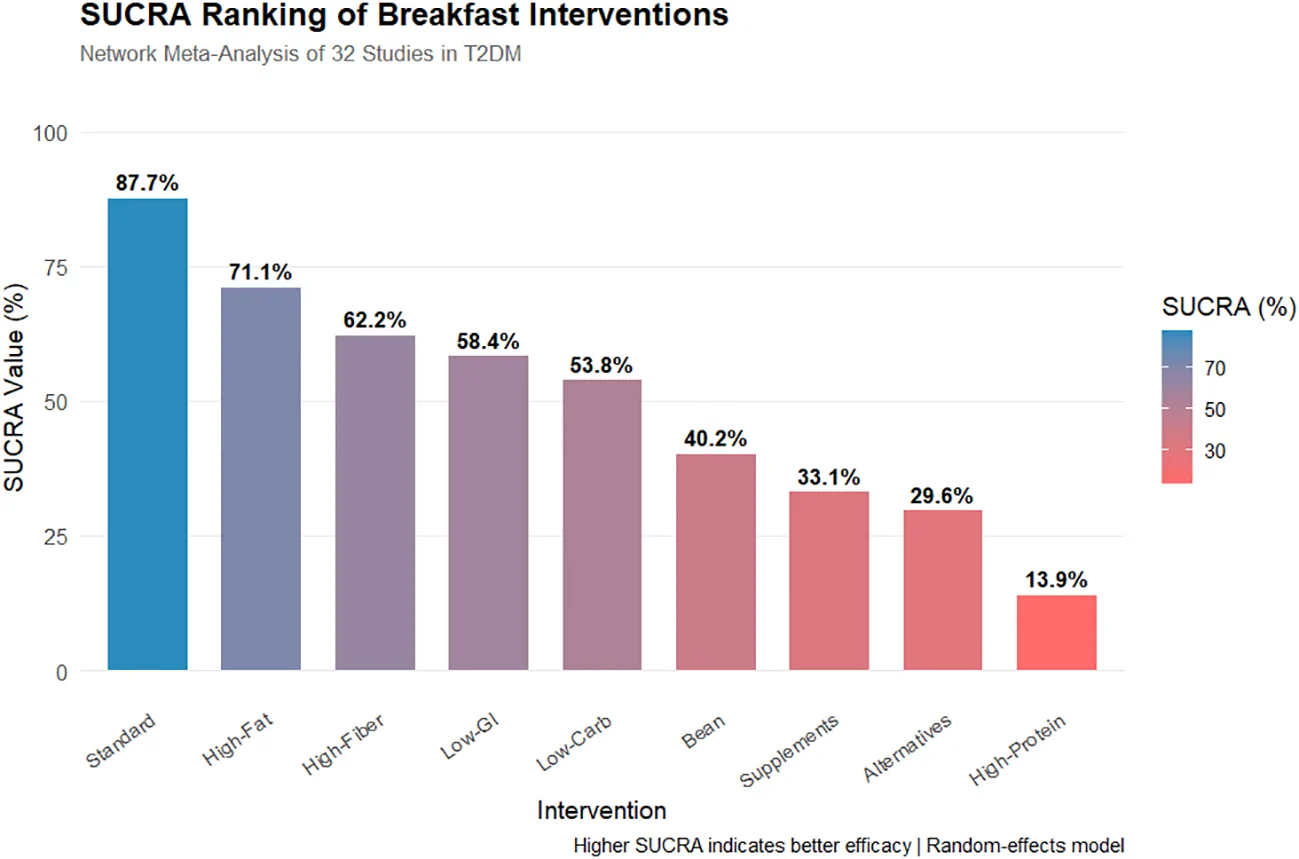
Ranking of SUCRA values for different interventions (%) in T2DM patients.

To determine the optimal breakfast macronutrient composition for glycemic management in T2DM, a network meta-analysis of 32 studies was performed to calculate SUCRA probabilities. According to the random-effects model (**Figure 11**), the Standard Breakfast (control diet) ranked first with a SUCRA value of 87.7%, indicating the highest probability of being the most efficacious intervention. Among the experimental dietary strategies, the High-Fat breakfast (SUCRA = 71.1%) and the High-Fiber breakfast (SUCRA = 62.2%) ranked as the second- and third-most effective interventions.

Intermediate rankings were observed for Low-GI (58.4%), Low-Carb (53.8%), and Bean-based breakfasts (40.2%). In contrast, Supplements (33.1%), Alternatives (29.6%), and High-Protein breakfasts (13.9%) were ranked as the least effective interventions.

### Trim-and-fill sensitivity analysis for publication bias

Given the statistically significant Egger’s test (*P* = 0.026) and visual asymmetry observed in the funnel plot, we performed a trim-and-fill sensitivity analysis to evaluate the potential impact of publication bias on the pooled effect estimate.

Importantly, although the trim-and-fill adjustment increased the magnitude of the negative effect estimate, the direction and statistical significance of the pooled result remained unchanged, with the 95% confidence interval still entirely excluding the line of null effect. This suggests that, while there is evidence of funnel plot asymmetry (likely driven by small-study effects and a lack of published null or positive findings), the overall conclusion that modified breakfast macronutrient compositions significantly improve glycemic outcomes in T2DM patients remains relatively robust to potential publication bias.

### GRADE certainty of evidence and clinical recommendations

GRADE certainty of evidence and recommendations for different breakfast interventions are summarized in **Table 2**.

High-Protein Breakfasts (High certainty): Pooled analysis (2 studies, n=115) showed a significant reduction in HbA1c (MD: -0.50%, 95% CI: -0.56 to -0.44; *I*^2^ = 0%), exceeding the MCID of 0.3–0.4%. Consequently, a Strong recommendation was made.

High-Fiber Breakfasts (Moderate certainty): Reduced 2h postprandial glucose (MD: -2.10 to -2.46 mmol/L) but showed a modest HbA1c effect (MD: -0.20%). We issued a Weak-to-moderate recommendation as an adjunctive strategy.

Breakfast Alternatives (Moderate certainty): Consistently reduced AUC and glycemic peaks, though definitions varied and daily mean glucose effects were small. A Weak-to-moderate recommendation was given, emphasizing individualization.

Low-GI Breakfasts (Moderate certainty): Pooled HbA1c showed a non-significant trend (MD: -0.21%, 95% CI: -0.54 to +0.12; *I*^2^ = 0%, 2 studies). The CI crossing the null led to a Weak recommendation for selected patients.

Supplements (Low certainty): The effect on 2h glucose was imprecise (MD: -1.06 mmol/L, 95% CI: -2.31 to +0.19) with high heterogeneity (*I*^2^ = 71%). No recommendation could be made due to insufficient evidence.

### Assessment of publication bias

**Figure 12:**
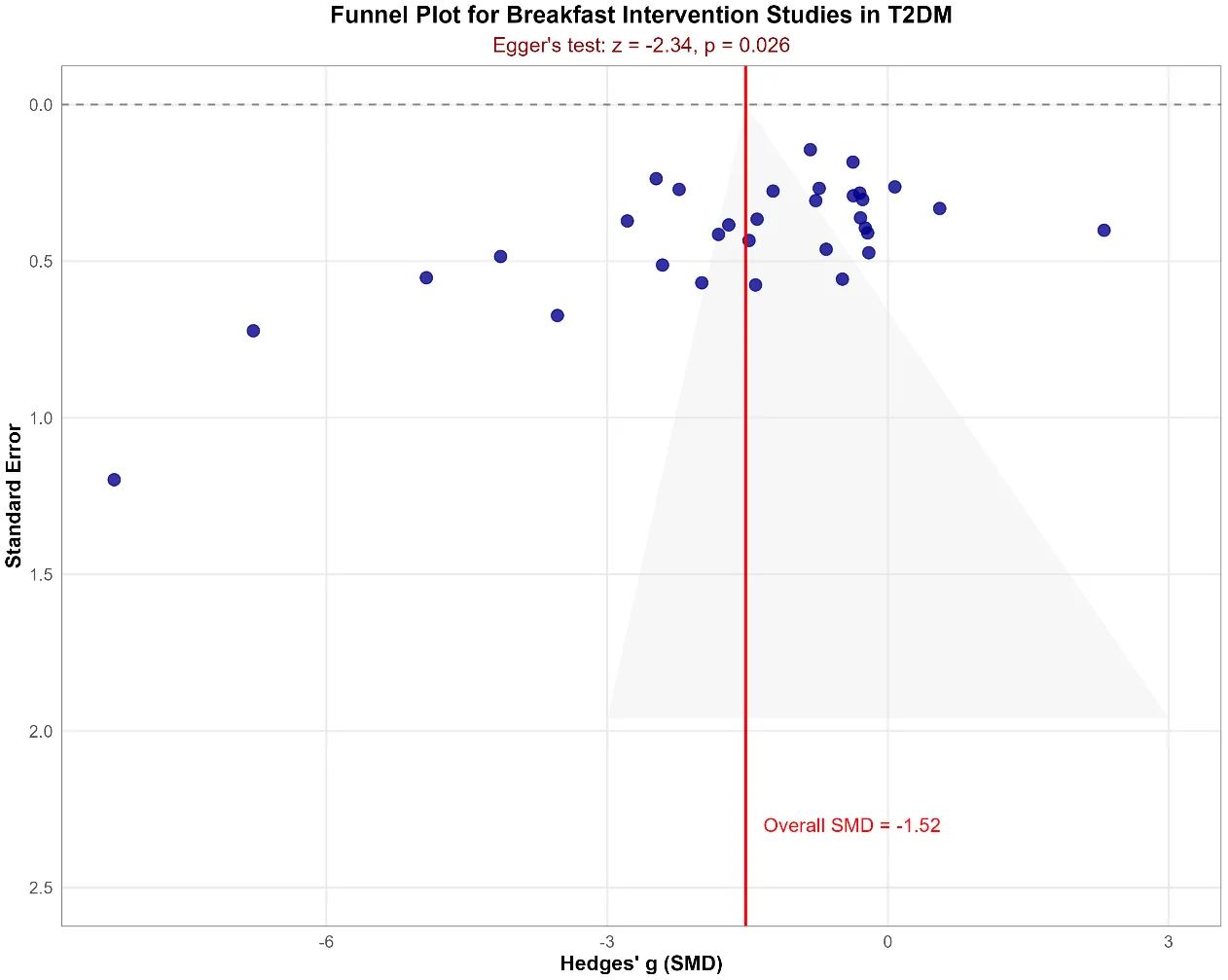
Funnel Ppot with egger's test.

To evaluate potential publication bias, we constructed a funnel plot and performed Egger’s linear regression test. Visual inspection of the funnel plot revealed an asymmetrical distribution, with a notable clustering of studies appearing to the left of the overall pooled estimate (SMD = -1.52, marked by the vertical red line) (**Figure 12**).

The funnel plot indicates a specific deficit of smaller studies reporting positive effect sizes (to the right of the red line). This pattern implies that studies showing unfavorable or null effects of breakfast interventions on glycemic control may be missing from the published literature. While trim-and-fill analysis could be considered to adjust for this bias, the presence of significant Egger’s test results underscores that our summary estimates may be overestimating the true efficacy of the interventions. Therefore, our findings should be interpreted with caution in light of the identified potential publication bias.

The methodological quality of the 37 included randomized controlled trials (RCTs) was assessed using the Cochrane Risk of Bias tool 2.0 (RoB 2.0). The overall risk of bias across the studies was mixed, with no study assessed as having an overall “Low risk” of bias. Specifically, 3 studies (8.1%) were rated as having an overall “High risk” of bias (Li Wen-hui, 2008; Jenkins DJA, 2002; and Daniela R. Lobos 2017, although Lobos was rated as “Some concerns” overall based on your table, please double-check; the clear High-risk ones are Wen-hui and Jenkins), and the remaining 34 studies (91.9%) were assessed as having “Some concerns” regarding overall bias. (**Table 3**).

**Table 3:** Results of the risk of bias assessment for included studies (Cochrane RoB 2.0).

No.	Study ID	D1	D2	D3	D4	D5	Overall
1	L. Lafrance 1998						
2	Janina Rynarzewski 2019						
3	Susan R Parsons 1984						
4	Dominique Chenon 1984						
5	Yasufumi Enyama 1982						
6	Di Li 2019						
7	Dirk J. Stenvers 2014						
8	Jace Schell 2017						
9	Daniela Jakubowicz 2017						
10	STEPHEN COLAGIURI 1986					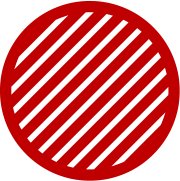	
11	Qing Xiong 2021						
12	Fernanda Duarte Moreira 2022						
13	Louise Rasmussen 2020			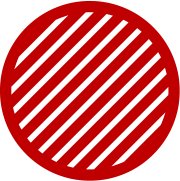			
14	Kevin C. Maki 2017						
15	Hadas Rachel Rabinovitz 2014						
16	Vikkie A Mustad 2020						
17	Sebastian Aberg 2020						
18	Stine Smedegaard 2025						
19	Sharon V Thompson 2012			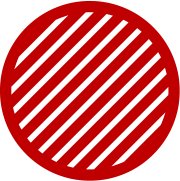			
20	Pardeep Pabla 2024						
21	Cl´audia M de Carvalho 2017						
22	Barbara F. Oliveira 2023						
23	Jimmy Chun Yu Louie 2012						
24	Sing Teang Kong 2024						
25	Daniela R. Lobos 2017	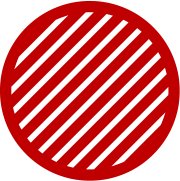					
26	Li Wen-hui 2008	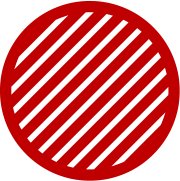					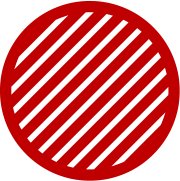
27	Maria N. Ballesteros 2015						
28	Alain Golay 1992						
29	Janani Narayanan 2016						
30	Patricia Lopez-Romero 2014						
31	Christine A. Clark 2006						
32	M. Kabir 2003						
33	Karma L. Pearce 2008						
34	Natalie C. Ward 2020						
35	David J. A. Jenkins 2002		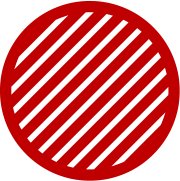	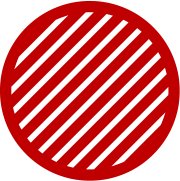			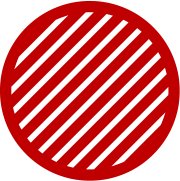
36	Pimpananut Sridonpai 2021						
37	Eva Pedersen 2016						


 Low risk.


 Some concerns.

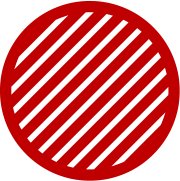
 High risk.

D1 Randomisation process.

D2 Deviations from the intended interventions.

D3 Missing outcome data.

D4 Measurement of the outcome.

D5 Selection of the reported result.

## Discussion

This systematic review and meta-analysis, comprising 37 randomized controlled trials across 1,030 patients with type 1 (T1DM), type 2 (T2DM), and gestational diabetes mellitus (GDM), comprehensively evaluated the impact of breakfast macronutrient composition on glycemic control. The primary pairwise meta-analysis demonstrated that modified breakfast interventions significantly reduced postprandial glucose excursions (MD: -0.52 mmol/L) and HbA1c levels (MD: -0.32%) compared with standard breakfasts. However, considerable heterogeneity (*I*^2^ = 93.7%) and notable discrepancies between pairwise and network meta-analytic rankings necessitate a nuanced interpretation of the optimal dietary strategy.

The findings of this study are generally consistent with those of previously published meta-analyses (48). Previous studies have shown that a high-fiber diet can significantly improve glycemic control in patients with type 2 diabetes, reducing HbA1c by approximately 0.2–0.5% (49). A high-fiber breakfast lowers postprandial blood glucose peaks by increasing chyme viscosity, slowing gastric emptying, and reducing glucose absorption (50). Soluble dietary fiber (such as pectin and guar gum) is particularly effective at lowering blood glucose levels (51).

Among all dietary strategies, high-protein breakfasts (enriched with eggs or whey protein) emerged as the most robust intervention in pairwise meta-analyses and were rated with High GRADE certainty. The observed pooled reduction in HbA1c (-0.50%) exceeded the clinically important threshold of 0.3%–0.4%, a finding that is particularly meaningful for long-term diabetes management. This effect is likely attributable to the enhanced satiety, increased thermogenesis, and reduced subsequent carbohydrate intake associated with protein-rich meals (4, 52).

High-fiber and plant-based breakfasts also demonstrated significant clinical value, particularly in lowering 2-hour postprandial glucose (MD: -2.10 to -2.46 mmol/L). However, their HbA1c-lowering effect was modest (-0.20%), suggesting that fiber’s primary benefit lies in attenuating acute glycemic peaks rather than in durable long-term control (48). Conversely, Low-GI breakfasts showed a favorable but statistically non-significant trend for HbA1c (MD: -0.21%, crossing the null), indicating that GI alone may be insufficient to drive meaningful clinical changes without concomitant modification of protein or fiber content.

An intriguing and unexpected finding from our network meta-analysis (NMA) was the SUCRA ranking, which placed Standard Breakfast (87.7%) as the most likely best intervention, while High-Protein breakfast (13.9%) ranked lowest. This apparent paradox can be explained by the specific network geometry and heterogeneity in outcome assessment across the included studies. First, the star-like network structure lacked closed loops, meaning most evidence relied on direct comparisons rather than leveraging indirect corrections. Second, and more critically, the NMA pooled a variety of glycemic outcomes—including fasting glucose, HbA1c, AUC, and postprandial peaks—into a single model. Because the clinical significance of a 0.5% HbA1c reduction differs markedly from that of a 5 mmol/L AUC change, the NMA may have inadvertently masked the specific benefits of acute interventions (such as high-protein) while favoring baseline-control diets. This finding emphasizes that SMD- or MD-based rankings from heterogeneous outcome pools should be interpreted with caution and should not override the clinically specific pairwise evidence, particularly for endpoints like postprandial glycemic excursions.

The extreme heterogeneity observed in both the AUC/iAUC subgroups (ranging from 86% to 94.1%) and the duration subgroups (*I*^2^ > 80%) largely stems from the lack of standardized methodology for assessing postprandial responses. Our subgroup analysis by measurement type confirmed that varying time windows (0–120min, 0–180min, 24 h) and using absolute AUC versus incremental iAUC yielded markedly different effect magnitudes. The leave-one-out sensitivity analysis for the iAUC cohort indicated that the overall effect was robust (not driven by a single outlying study); however, the persistence of *I*^2^ > 55% after removing any single study suggests that the variability is inherent to the heterogeneous designs, populations, and intervention types used across trials.

Subgroup findings for GDM and T1DM populations, though limited by small sample sizes, provide important preliminary insights. In T1DM, macronutrient manipulation may benefit even insulin-dependent patients by smoothing postprandial spikes. In GDM, longer-term studies measuring TIR and mean glucose failed to demonstrate significant benefits. This indicates that GDM patients may require more sustained or different dietary patterns to achieve durable glycemic stability than acute breakfast modifications alone (53, 54).

In addition to the direct impact of breakfast’s macronutrient composition on blood glucose levels, potential interactions with concomitant antidiabetic medications also warrant consideration. The diabetic patients included in the trials received a variety of treatment regimens, including metformin, insulin, sulfonylureas, and GLP-1 receptor agonists; there was significant heterogeneity across studies in drug type, dosage, and treatment duration. Importantly, none of the original randomized controlled trials (RCTs) conducted pre-specified subgroup analyses based on baseline medication use; therefore, it is not possible to assess whether the glycemic responses observed with the modified breakfast differed across different baseline treatment regimens. Consequently, the results of this network meta-analysis should be interpreted as reflecting the average effect in a heterogeneous population of patients on mixed medication regimens, and their generalizability to specific medication subgroups remains unclear.

## Future research and clinical implications

Despite the limitations, our results support a pragmatic, individualized approach to recommending breakfast interventions for patients with diabetes. Given the robust pairwise evidence and high GRADE certainty, high-protein breakfasts should be a primary dietary recommendation for T2DM patients aiming for sustained HbA1c reduction. For patients with marked postprandial hyperglycemia, high-fiber or low-GI breakfasts remain valuable adjuncts.

## Limitations

### High statistical heterogeneity and clinical variability

The most prominent limitation is the consistently high statistical heterogeneity observed across nearly all primary and subgroup analyses (overall *I*^2^ = 93.7%; AUC/iAUC subgroups *I*^2^ > 80%). While we explored potential sources of heterogeneity through prespecified subgroup analyses based on intervention duration and outcome assessment methods, the residual heterogeneity remains substantial. This variability is likely attributable to the wide diversity in baseline patient characteristics (e.g., disease duration, insulin use, baseline HbA1c), differences in the exact composition and dosage of the macronutrient interventions (e.g., type of protein, fiber source, or carbohydrate quality), and the lack of standardized postprandial assessment protocols across studies.

### Assessment of publication bias

The statistical significance of Egger’s test (*P* = 0.026) and the visual asymmetry observed in the funnel plot indicate potential publication bias or small-study effects. The deficit of smaller studies reporting null or positive results suggests that our summary effect estimates may slightly overestimate the true clinical efficacy of certain breakfast interventions. Although the trim-and-fill sensitivity analysis demonstrated that the overall direction and statistical significance of the pooled estimates remained robust after imputing potentially missing studies, the exact magnitude of the effects should still be interpreted with caution.

### Heterogeneity of outcome metrics and units across studies

Our meta-analysis aimed to synthesize diverse glycemic endpoints (e.g., HbA1c, FPG, 2hBS, absolute AUC, iAUC) using standardized mean differences (SMDs) to account for differing units and scales. However, combining acute postprandial indicators (which measure peak excursions) with chronic glycemic markers (which measure average glucose over weeks) poses clinical and statistical challenges. While SMD is a useful statistical tool for combining these outcomes, it does not always convey clinically interpretable magnitudes. Furthermore, the AUC/iAUC measurements varied widely in their recording durations (from 1 hour to 24 hours) across major subgroups.

### Limited sample sizes for T1DM and GDM subgroups

For the type 1 diabetes (T1DM) and gestational diabetes mellitus (GDM) populations, the evidence base is critically limited. The T1DM analysis relied on only two small RCTs (totaling 30 participants), and the GDM analysis comprised merely three RCTs, with extremely inconsistent outcome measures (TIR vs. mean glucose vs. iAUC).

### Limitations of drug-diet interactions

The included studies did not stratify or analyze results by the antidiabetic medications participants were taking (e.g., metformin, insulin, GLP-1 receptor agonists). Given that the mechanisms of action of these medications differ from those of dietary interventions but also partially overlap, the observed effect sizes may be subject to confounding due to differences in medication characteristics across studies. This precludes a meaningful assessment of synergistic or antagonistic interactions between medications and diet, and also limits the applicability of these findings to patients following specific treatment regimens.”

## Conclusions

In conclusion, the macronutrient and dietary fiber composition of breakfast modifications—particularly high-protein options—is a clinically useful and evidence-supported tool for managing diabetes. To translate these findings into robust clinical practice guidelines, future well-designed, long-term RCTs must adopt standardized postprandial assessment protocols, ensure strict isocaloric control, and evaluate hard clinical outcomes in both understudied populations and diverse ethnic groups.

## Data Availability

The original contributions presented in the study are included in the article/**Supplementary Material**. Further inquiries can be directed to the corresponding authors.
