# Supplementary material for: Effects of breakfast macronutrient composition on postprandial glycemic control in patients with diabetes mellitus: a systematic review and meta-analysis of randomized controlled trials
Source: Front Endocrinol (Lausanne). 2026 Aug 17;17:1859725. doi: 10.3389/fendo.2026.1859725 (PMC13526540; doi:10.3389/fendo.2026.1859725)
Supplement: Supplementary file 1 [file DataSheet1.docx]

**Table S1. Search Strategy**

| **Database** | **Number of articles retrieved** |
| --- | --- |
| **Web of Science** | 4003 |
| ("breakfast" OR "morning meal") AND ("macronutrient composition" OR "macronutrient order" OR "carbohydrate last" OR "meal sequence" OR "nutrient preload*") AND ("postprandial glycemic control" OR "postprandial glucose" OR "glycemic excursion" OR "blood glucose" OR "glycemic response") AND ("diabetes mellitus" OR "type 1 diabetes" OR "type 2 diabetes" OR "gestational diabetes" OR "T1DM" OR "T2DM" OR "GDM") AND ("systematic review" OR "meta-analysis" OR "randomized controlled trial" OR "RCT") |  |
| **Pubmed** | 230 |
| ("breakfast" OR "morning meal"[tiab]) AND ("macronutrient composition"[tiab] OR "meal sequence"[tiab] OR "carbohydrate last"[tiab] OR "nutrient preload"[tiab]) AND ("postprandial glycemic control"[tiab] OR "postprandial glucose"[tiab] OR "glycemic excursion"[tiab] OR "blood glucose"[tiab] OR "glycemic response"[tiab]) AND ("diabetes mellitus"[mh] OR "type 1 diabetes"[tiab] OR "type 2 diabetes"[tiab] OR "gestational diabetes"[tiab] OR T1DM[tiab] OR T2DM[tiab] OR GDM[tiab]) AND ("systematic review"[pt] OR "meta-analysis"[pt] OR "randomized controlled trial"[pt] OR RCT[tiab]) |  |
| **Cochrane** | 1266 |
| #1 MeSH descriptor: [Diabetes Mellitus] explode all trees  #2 (diabetes OR "diabetes mellitus" OR T1DM OR T2DM OR GDM OR "type 1 diabetes" OR "type 2 diabetes" OR "gestational diabetes"):ti,ab,kw  #3 #1 OR #2  #4 MeSH descriptor: [Breakfast] explode all trees  #5 (breakfast OR "breakfast meal" OR morning meal):ti,ab,kw  #6 #4 OR #5  #7 MeSH descriptor: [Dietary Carbohydrates] explode all trees  #8 MeSH descriptor: [Dietary Proteins] explode all trees  #9 MeSH descriptor: [Dietary Fats] explode all trees  #10 MeSH descriptor: [Dietary Fiber] explode all trees  #11 (macronutrient* OR carbohydrate* OR protein* OR fat* OR fiber OR fibre OR "glycemic index" OR "low GI" OR "high fiber" OR "low carbohydrate" OR "whey protein"):ti,ab,kw  #12 #7 OR #8 OR #9 OR #10 OR #11  #13 MeSH descriptor: [Blood Glucose] explode all trees  #14 MeSH descriptor: [Glycated Hemoglobin] explode all trees  #15 (postprandial OR "post-prandial" OR glucose OR glycemia OR glycaemia OR HbA1c OR "blood glucose" OR "glycemic control"):ti,ab,kw  #16 #13 OR #14 OR #15  #17 #3 AND #6 AND #12 AND #16 |  |
| **Embase** | 6958 |
| ('diabetes mellitus'/exp OR 'diabetes mellitus' OR 'type 1 diabetes'/exp OR 'type 1 diabetes' OR 'type 2 diabetes'/exp OR 'type 2 diabetes' OR 'gestational diabetes'/exp OR 'gestational diabetes' OR T1DM OR T2DM OR GDM)  AND  ('breakfast'/exp OR breakfast OR 'morning meal' OR 'first meal')  AND  ('macronutrient'/exp OR macronutrient* OR 'carbohydrate'/exp OR carbohydrate* OR 'protein'/exp OR protein* OR 'fat'/exp OR fat* OR 'dietary fiber'/exp OR fiber OR fibre OR 'glycemic index'/exp OR 'glycemic index' OR 'low gi' OR 'high fiber' OR 'low carbohydrate' OR 'whey protein'/exp OR 'whey protein' OR legume* OR bean*)  AND  ('postprandial state'/exp OR 'postprandial state' OR postprandial OR 'post-prandial' OR 'blood glucose'/exp OR 'blood glucose' OR glucose OR glycemia OR glycaemia OR 'hemoglobin a1c'/exp OR 'hemoglobin a1c' OR HbA1c OR 'glycemic control' OR AUC OR iAUC OR 'area under the curve') |  |
| **ScienceDirect** | 123 |
| (diabetes OR "diabetes mellitus" OR T1DM OR T2DM OR GDM OR "type 1 diabetes" OR "type 2 diabetes" OR "gestational diabetes")  AND  (breakfast OR "morning meal" OR "first meal")  AND  (macronutrient* OR carbohydrate* OR protein* OR fat* OR fiber OR fibre OR "glycemic index" OR "low gi" OR "high fiber" OR "low carbohydrate" OR "whey protein" OR legume* OR bean*)  AND  (postprandial OR "post-prandial" OR glucose OR glycemia OR glycaemia OR HbA1c OR "blood glucose" OR "glycemic control" OR AUC OR iAUC OR "area under the curve") |  |

**Table S2.** The inclusion and exclusion criteria for screening articles

| **Inclusion criteria** | **Reason** |
| --- | --- |
| (1) The language type was English | English, one of the most common international languages, was chosen to facilitate the researcher's screening and study. |
| (2) The literature type was research articles | Ensure that articles included in the analysis contain the information the researcher needs, such as specific research outcomes. |
| (3) The data source was Web of Science or PubMed | Web of Science is an authoritative, comprehensive database, and PubMed is the leading database in the field of biomedicine. Higher quality of articles. |
| **Exclusion criteria** | **Reason** |
| (1) duplicate article | Make sure to get a realistic number of articles. |
| (2) literature for which full text is not available, such as conference abstracts, etc. | Insufficient or missing literature data for systematic reviews. |
| (3) unrelated to the topic | It is completely unrelated to the study topic and cannot be analyzed. |

**Figure S1.** Subgroup Meta-analysis of Randomized Controlled Trials Comparing Intervention Breakfasts vs Standard Breakfasts on Postprandial Glycemic Control, Stratified by Diabetes Mellitus Type (T1DM, T2DM, GDM).

**Figure S2.** Forest plot of subgroup analysis by intervention duration for the effect of breakfast macronutrient composition on postprandial glycemic control in patients with diabetes mellitus.

**Figure S3.** Subgroup comparison of baseline characteristics (age, HbA1c, diabetes type)
